# IF1 ablation prevents ATP synthase oligomerization, enhances mitochondrial ATP turnover and promotes an adenosine-mediated pro-inflammatory phenotype

**DOI:** 10.1038/s41419-023-05957-z

**Published:** 2023-07-12

**Authors:** Sonia Domínguez-Zorita, Inés Romero-Carramiñana, Fulvio Santacatterina, Pau B. Esparza-Moltó, Carolina Simó, Araceli del-Arco, Cristina Núñez de Arenas, Jorge Saiz, Coral Barbas, José M. Cuezva

**Affiliations:** 1 Departamento de Biología Molecular, Centro de Biología Molecular Severo Ochoa, Consejo Superior de Investigaciones Científicas-Universidad Autónoma de Madrid (CSIC-UAM), 28049 Madrid, Spain; 2 Centro de Investigación Biomédica en Red de Enfermedades Raras (CIBERER) ISCIII, Madrid, Spain; 3 Instituto de Investigación Hospital 12 de Octubre, Universidad Autónoma de Madrid, Madrid, Spain; 4 Molecular Nutrition and Metabolism, Institute of Food Science Research (CIAL, CSIC-UAM), 28049 Madrid, Spain; 5 Facultad de Ciencias Ambientales y Bioquímica, Universidad de Castilla la Mancha, Toledo, 45071 Spain; 6 Centro Regional de Investigaciones Biomédicas, Unidad Asociada de Biomedicina, Toledo, 45071 Spain; 7 Centre of Metabolomics and Bioanalysis (CEMBIO), Department of Chemistry and Biochemistry, School of Pharmacy, Universidad San Pablo-CEU, CEU Universities, Urbanización Montepríncipe, 28660 Boadilla del Monte, Madrid, Spain

**Keywords:** Energy metabolism, Metabolomics, Proteomics, Chronic inflammation

## Abstract

ATPase Inhibitory Factor 1 (IF1) regulates the activity of mitochondrial ATP synthase. The expression of IF1 in differentiated human and mouse cells is highly variable. In intestinal cells, the overexpression of IF1 protects against colon inflammation. Herein, we have developed a conditional IF1-knockout mouse model in intestinal epithelium to investigate the role of IF1 in mitochondrial function and tissue homeostasis. The results show that IF1-ablated mice have increased ATP synthase/hydrolase activities, leading to profound mitochondrial dysfunction and a pro-inflammatory phenotype that impairs the permeability of the intestinal barrier compromising mouse survival upon inflammation. Deletion of IF1 prevents the formation of oligomeric assemblies of ATP synthase and alters cristae structure and the electron transport chain. Moreover, lack of IF1 promotes an intramitochondrial Ca^2+^ overload in vivo, minimizing the threshold to Ca^2+^-induced permeability transition (mPT). Removal of IF1 in cell lines also prevents the formation of oligomeric assemblies of ATP synthase, minimizing the threshold to Ca^2+^-induced mPT. Metabolomic analyses of mice serum and colon tissue highlight that IF1 ablation promotes the activation of de novo purine and salvage pathways. Mechanistically, lack of IF1 in cell lines increases ATP synthase/hydrolase activities and installs futile ATP hydrolysis in mitochondria, resulting in the activation of purine metabolism and in the accumulation of adenosine, both in culture medium and in mice serum. Adenosine, through ADORA2B receptors, promotes an autoimmune phenotype in mice, stressing the role of the IF1/ATP synthase axis in tissue immune responses. Overall, the results highlight that IF1 is required for ATP synthase oligomerization and that it acts as a brake to prevent ATP hydrolysis under in vivo phosphorylating conditions in intestinal cells.

## Introduction

The mitochondrial ATP synthase is the enzyme that synthetizes cellular ATP by oxidative phosphorylation (OXPHOS) [1] and plays a structural role in the inner membrane by forming dimers and oligomers to shape cristae at its rims [2]. Moreover, the ATP synthase is a relevant component of the signaling hub of mitochondria that participates both in nuclear reprogramming to allow cellular adaption to changing cues [3, 4] or in the execution of cell death by its structural and functional implication in permeability transition (mPT) [5–8].

The ATPase inhibitory factor 1 (IF1) is a physiological inhibitor of the ATP synthase [9, 10] that is encoded in the nuclear *ATP5IF1* gene and exerts its inhibitory activity by binding to the catalytic interface in the F_1_ domain of the enzyme [11]. IF1 is not ubiquitously expressed, and large differences in IF1 expression levels exist between differentiated cells of human and mouse tissues [12]. Moreover, it seems that IF1 plays a role in the oligomerization of the ATP synthase, as recently shown by cryo-EM structures of mammalian ATP synthases [13, 14]. In these studies, IF1 dimers act as staples linking two adjacent antiparallel dimers of ATP synthases to form inactive tetramers of the enzyme [13, 14]. These findings support that IF1 interacts with the ATP synthase under mitochondrial phosphorylating conditions, which is in agreement with biochemical and functional data in mouse models of the loss and gain of function of IF1 [15–20].

Recent findings emphasize the potential implication of IF1 in human pathophysiology, such as in cancer [7, 21], diabetes [22–24], ischemia [25], cardiovascular [20, 26] and neurodegenerative [18, 27] diseases. In this regard, IF1 plays an anti-apoptotic role in protecting cells from death by different mechanisms [7, 15, 28–30]. ATP synthase and its oligomeric assemblies have been proposed to contribute to the formation of mitochondrial permeability transition pores (PTP) [5, 6, 8, 14, 31–35]. Hence, the role of IF1 in cell death relies on its structural and functional implication in the regulation of ATP synthase. However, it remains to be investigated the role of IF1 in PTP formation in vivo in mouse models of human pathophysiology due to the tissue-specific expression of IF1.

Interestingly, a transgenic mouse model conditionally overexpressing IF1 in the intestinal epithelium showed that IF1 switched the immune system of the tissue towards an anti-inflammatory phenotype [17], highlighting a link between the IF1/ATP synthase axis and the immune response. Herein, we have developed a conditional intestine-specific IF1 *knockout* mouse model (IF1-KO) and show that its ablation prevents ATP synthase oligomerization and triggers a profound alteration of mitochondrial structure and function. IF1 absence activates a futile cycle of ATP hydrolysis in mitochondria, greatly increasing the production and accumulation of the immunometabolite adenosine. These changes result in an altered permeability of the epithelial barrier facilitating bacterial infection and a hampered adenosine-mediated tissue immune response, stressing the relevance of the IF1/ATP synthase axis in tissue immune responses.

## Results

### A conditional IF1 knockout mouse in the intestinal epithelium

**Fig. 1:**
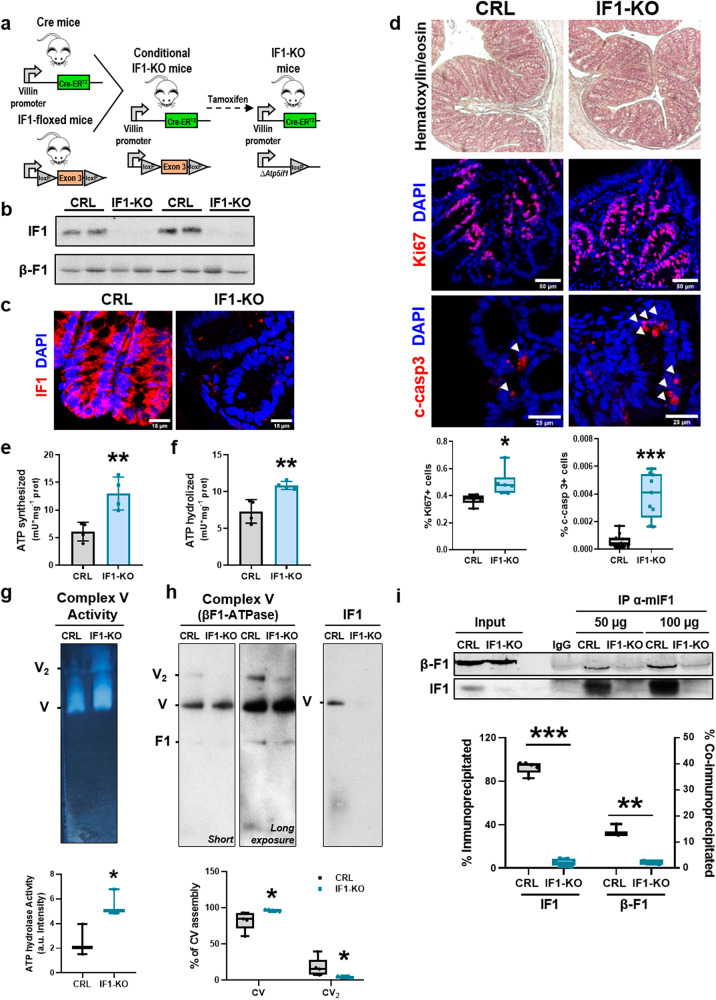
IF1 affects oligomerization and activity of ATP synthase. **a** IF1-KO mice were obtained by breeding the villin-Cre-ER^T2^ line with the IF1-floxed mouse line after tamoxifen administration. **b** Representative blot of IF1 and β-F1-ATPase expression in four different colon samples of control (CRL) and IF1-KO mice. **c** Representative immunofluorescence of IF1 expression (red) in the colon. DAPI (blue) stained nuclei. **d** Representative hematoxylin-eosin and immunofluorescence to illustrate proliferation (Ki67, red) and apoptotic cell death (c-casp3, red) in colon of CRL and IF1-KO mice. DAPI (blue) stained nuclei. Box-and-whisker plots show the percentage of Ki67+ and c-casp3+ cells per total nuclei (*n* = 5). **e**, **f** Synthetic (**e**) and hydrolytic (**f**) activities of ATP synthase in colon mitochondria of CRL and IF1-KO mice. Mean ± SEM (*n* = 4). **g** ATP hydrolytic activity of complex V in CN-PAGE gels. The box-and-whisker plot shows the quantification of ATP hydrolysis in colon mitochondria of CRL and IF1-KO mice (*n* = 3). **h** BN-PAGE of complex V in colon mitochondria from CRL and IF1-KO mice. β-F1-ATPase (complex V) and IF1 blots are shown. The box-and-whisker plot shows the percentage of complex V (CV) in monomeric (V) or dimeric (V_2_) form (*n* = 5). **i** Co-immunoprecipitation of β-F1-ATPase and IF1 from colon mitochondria of CRL and IF1-KO mice (*n* = 3) using an antibody against murine IF1. Co-immunoprecipitated β-F1-ATPase (β-F1) was revealed with anti-β-F1 labeled with Cy5. Immunoprecipitations were performed with an input of 50 or 100 µg of protein. Nonspecific mouse immunoglobulin (IgG) is included as a control. Box-and-whisker plots show the percentage of IF1 immunoprecipitated and the percentage of ATP synthase co-immunoprecipitated. *p ≤ 0.05, **p ≤ 0.01, ***p ≤ 0.001 when compared by Student’s *t*-test. See also Supplementary Fig. S1.

A conditional *Atp5if1* knockout (IF1-KO) mice was developed by breeding villin-Cre-ER^T2^ mice [36] with the IF1-floxed mice [18] (Fig. 1a). IF1-KO mice were obtained by activation of Cre recombinase after the administration of tamoxifen, triggering deletion of exon 3 of *Atp5if1* (Fig. 1a). After 2 weeks of tamoxifen administration, IF1-KO mice showed no IF1 expression in cells of the intestinal epithelium when compared to littermate villin-Cre-ER^T2^ mice (Fig. 1b, c). No gross changes in the length (Supplementary Fig. S1a) or histology of the colon (Fig. 1d) were observed in IF1-KO mice when compared to controls. However, the proliferation and apoptotic death of colonocytes were significantly augmented in IF1-KO mice (Fig. 1d and Supplementary Fig. S1b). The effect of IF1 ablation in cellular proliferation seems to be cell-type dependent [7, 21]. No evidence of autophagic induction was observed in the colon of IF1-KO mice (Supplementary Fig. S1c).

### IF1 affects the activity and oligomerization of the ATP synthase

Ablation of IF1 promoted a significant increase in both the ATP synthetic (Fig. 1e) and hydrolytic (Fig. 1f) activities of ATP synthase as determined in isolated mitochondria of the colon, suggesting that IF1 is bound to and inhibits a fraction of ATP synthase under physiological conditions. Consistently, assessment of the hydrolytic activity of the enzyme in CN gels confirmed that ablation of IF1 results in a sharp increase in in-gel activity of the enzyme (Fig. 1g). BN gels indicated that deletion of IF1 diminished the oligomeric assemblies of the ATP synthase and confirmed that a fraction of the enzyme co-fractionates with IF1 in control mice (Fig. 1h). Consistently, immunoprecipitation of IF1 from isolated mitochondria only promoted the co-immunoprecipitation of a significant fraction of β-F1-ATPase (~15%) in control mice (Fig. 1i). These results further support the existence of a relevant fraction of IF1-bound and inhibited ATP synthase in colon mitochondria.

### Ablation of IF1 alters the expression and activity of the respiratory chain

**Fig. 2:**
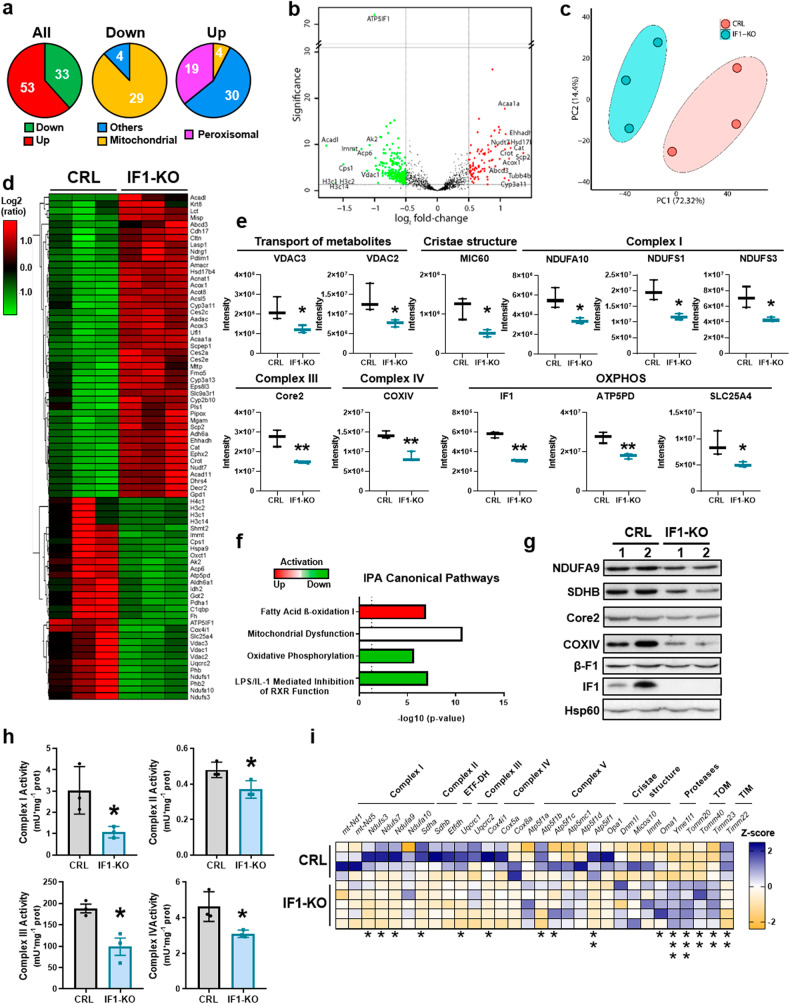
Ablation of IF1 affects the expression and activity of the respiratory chain. TMTsixplex proteomic analysis of isolated colon mitochondria from control (CRL) and IF1-KO mice (*n* = 3). **a** Diagram representing the total amount of proteins increased or decreased in mitochondria of IF1-KO mice. Middle and right diagrams show differentially expressed proteins as a function of their cellular location. **b** Volcano plot showing differentially expressed proteins between the two genotypes. **c** Principal component (PC) analysis showing the two genotypes. **d** Heat Map representation showing decreased (green) and increased (red) proteins in mitochondria of IF1-KO mice. **e** Box-and-whisker plots showing the expression levels of mitochondrial proteins involved in the transport of metabolites (VDAC2/3), cristae structure (MIC60), subunits of complex I (NDUFA9, NDUFS1/3), complex III (Core2), complex IV (COXIV) and OXPHOS (IF1, ATP5D, SLC25A4). **f** Ingenuity Pathway Analysis (IPA) of the predicted activation or inhibition of different canonical pathways in mitochondria of IF1-KO mice. **g** Representative blots of the expression of subunits of complexes I (NDUFA9), II (SDHB), III (Core2), IV (COXIV) and V (β-F1 and IF1) in two independent preparations of colon mitochondria (*n* = 4). Hsp60 is shown as loading control. **h** Histograms show the activities of complexes I, II, III, and IV. Mean ± SEM in intestine mitochondria of CRL and IF1-KO mice (*n* = 3). **i** Heat Map representation showing normalized *Z*-score levels of mRNA levels of genes encoding different mitochondrial proteins in the colon of CRL (*n* = 4) and IF1-KO mice (*n* = 5). *p ≤ 0.05, **p ≤ 0.01, ***p ≤ 0.001 when compared by Student’s *t*-test. Supplementary Figs. S1 and S2.

A proteomic analysis of crude mitochondrial preparations of IF1-KO and control mice identified 19 549 peptides, corresponding to 86 differentially expressed proteins (Fig. 2a, b), that clearly separated the two genotypes (Fig. 2c, d). Ablation of IF1 significantly reduced the expression of a very large number of mitochondrial proteins involved in the transport of metabolites (VDAC2/3), cristae structure (MIC60), the electron transport chain (NDUFS1/3, NDUFA10, Core2 and COXIV) and oxidative phosphorylation (SLC25A4, ATP5PD and IF1) (Fig. 2e). In contrast, it promoted the upregulation of peroxisomal β-oxidation proteins (Fig. 2a and Supplementary Fig. S1d). Ingenuity pathway analysis (IPA) of the proteome (Fig. 2f) predicted that IF1-KO mice had mitochondrial dysfunction, diminished oxidative phosphorylation and LPS-mediated inhibition of the immune response. In contrast, an enhanced fatty acid β-oxidation was predicted in IF1-KO mice (Fig. 2f), consistent with the upregulation of β-oxidation proteins (Fig. 2a and Supplementary Fig. S1d).

The diminished expression of proteins involved in the respiratory chain of IF1-KO mice was confirmed by blotting against relevant subunits of different respiratory complexes (Fig. 2g and Supplementary Fig. S1e) and by the reduction of the activity of respiratory complexes I, II, III and IV in mitochondria of IF1-KO mice (Fig. 2h). Gene expression analysis (Fig. 2i) suggested that the specific downregulation of respiratory complexes is exerted at a transcriptional level (Fig. 2i). However, we cannot exclude the participation of additional post-translational regulation of the electron transport chain by selective degradation of the proteins mediated by mitochondrial proteases. In fact, some of the inner membrane proteases are increased in IF1-KO mice (Fig. 2i and Supplementary Fig. S1f). Despite the profound alteration of the mitochondrial respiratory chain, we observed neither oxidative damage to mtDNA (Supplementary Fig. S2a) nor induction of the cellular and mitochondrial antioxidant system (Supplementary Fig. S2b, c). Likewise, no oxidative damage to cellular proteins was observed in IF1-KO mice when compared to controls (Supplementary Fig. S2d–f).

### Ablation of IF1 affects the structure of mitochondria

**Fig. 3:**
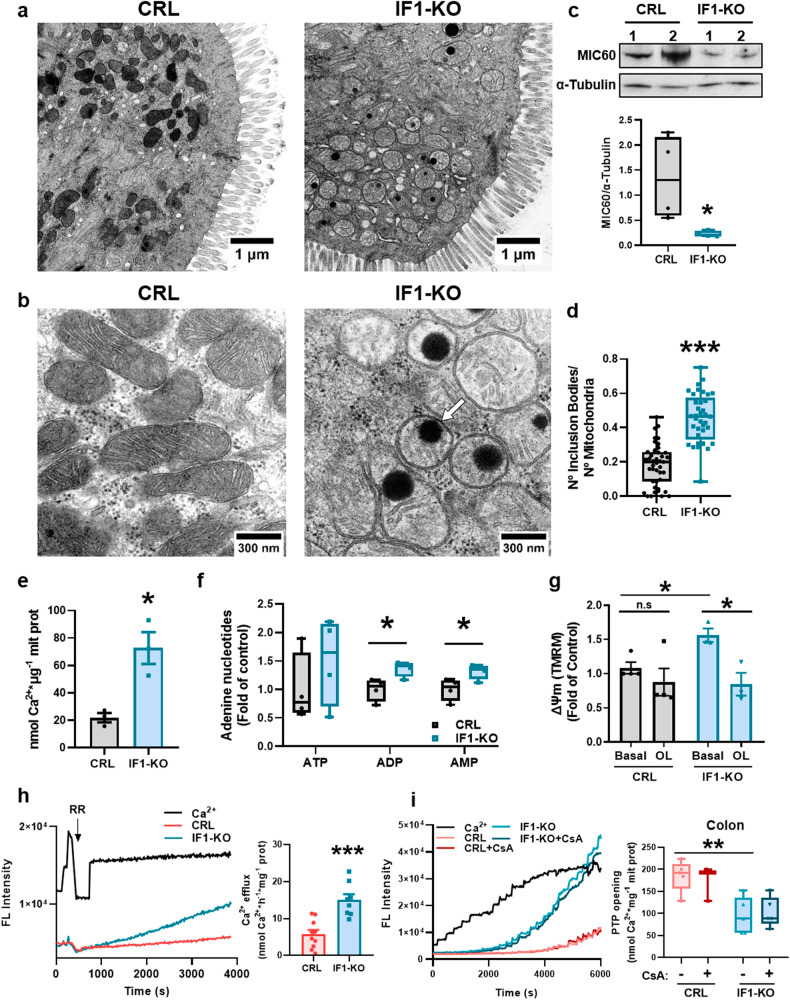
Ablation of IF1 results in altered mitochondrial structure and function. **a**, **b** Representative electron microscopy images of colonic epithelium. A white arrow in (**b**) shows an inclusion body. **c** Representative blot of MIC60 expression in two independent preparations of the colon of CRL and IF1-KO mice is shown. α-Tubulin is shown as a loading control. Box-and-whisker plot shows MIC60 expression relative to α-Tubulin (*n* = 4). **d** Box-and-whisker plots show the quantification of inclusion bodies per mitochondria (*n* = 37–43 mitochondria in 4 mice per genotype). **e** Histograms show mean ± SEM of the intramitochondrial Ca^2+^ content of CRL and IF1-KO mice (*n* = 3). **f** Box-and-whisker plots show adenine nucleotide content in colon mitochondria of CRL and IF1-KO mice (*n* = 4). **g** Histograms show mean ± SEM of mitochondrial membrane potential (∆Ψm) in colonocytes from CRL (*n* = 4) and IF1-KO (*n* = 3) mice. The effect of oligomycin (OL) is shown. **h** Ca^2+^ efflux rates in colon mitochondria. Left, representative traces of the fluorescence intensity of Ca^2+^-green after 3 pulses of 2 nmol Ca^2+^. The rate of Ca^2+^ efflux was recorded after the addition of 0.2 µM ruthenium red (RR) in mitochondria of CRL (*n* = 5 assayed in duplicate) and IF1-KO (*n* = 4 assayed in duplicate) mice. Right, histograms show the Ca^2+^ efflux rate mean ± SEM. **i** Ca^2+^ retention capacity (CRC) in colon mitochondria of CRL (*n* = 3–5) and IF1-KO (*n* = 5) mice. Left, representative traces of the fluorescence intensity of Ca^2+^-green in the presence or absence of cyclosporine A (CsA). Mitochondria were challenged with the addition of 2 nmol Ca^2+^. Right, box-and-whisker plots show the amount of Ca^2+^ required to induce PTP opening. *p ≤ 0.05, **p ≤ 0.01, ***p ≤ 0.001 when compared according to the Student’s *t*-test. See also Supplementary Fig. S3.

Electron microscopy analysis revealed that colon mitochondria of IF1-ablated mice are more circular, have shorter cristae and less electron density when compared to mitochondria in controls (Fig. 3a, b and Supplementary Fig. S3a–c). However, there were no relevant differences in the number of mitochondria per cell (Supplementary Fig. S3d), also confirmed by the lack of differences in mtDNA copy number (Supplementary Fig. S3e) and in the expression of mitochondrial transcription factor A (TFAM) (Supplementary Fig. S3f). The alteration of cristae structure in IF1-KO mice could result from diminished ATP synthase dimers (Fig. 1h) [2, 37] and other cristae-organizing proteins such as the MICOS complex (MIC60) (Figs. 2e, 3c). Other components affecting cristae structure and mitochondrial dynamics were not significantly affected (Supplementary Fig. S3g).

Remarkably, the mitochondria of IF1-ablated mice showed a significant increase in the number of electrondense inclusion bodies (Fig. 3b, d). The composition of this type of inclusion bodies is ascribed to amorphous calcium phosphate (ACP) aggregates [38, 39]. The formation of Ca-P deposits is stimulated by the intramitochondrial Ca^2+^ concentration, the mitochondrial membrane potential (∆Ψm) and the content of adenine nucleotides [40]. In agreement with this, we found that the intramitochondrial Ca^2+^ concentration (Fig. 3e), the content of adenine nucleotides (Fig. 3f) and ∆Ψm (Fig. 3g) were increased in colon mitochondria of IF1-KO, suggesting the dysregulation of Ca^2+^ transport in colon mitochondria of IF1-KO mice. However, no differences in the expression of the mitochondrial Ca^2+^ uniporter MCU, its regulatory subunit MiCU and in the short Ca^2+^-dependent mitochondrial carrier 1 (SCaMC-1) (Supplementary Fig. S3h) were observed.

### Ablation of IF1 alters mitochondrial Ca^2+^ homeostasis and PTP opening

Accumulation of Ca^2+^ in mitochondria could result from dysregulation of Ca^2+^ efflux pathways through the mitochondrial Na^+^/Ca^2+^ (NCLX) and H^+^/Ca^2+^ (NiCE) exchangers [41]. Determination of Ca^2+^ efflux in the presence of Ruthenium Red, an inhibitor of the activity of Ca^2+^ uniporter, revealed that Ca^2+^ efflux was significantly enhanced in mitochondria of IF1-KO mice (Fig. 3h), most likely to compensate for the excessive accumulation of intramitochondrial Ca^2+^ observed (Fig. 3e), and stressing the hyperactivation of Ca^2+^ metabolism in mitochondria of IF1-KO mice.

Consistent with the dysregulation of mitochondrial Ca^2+^ in IF1-KO mice, determination of the Ca^2+^ retention capacity (CRC) in colon mitochondria revealed that IF1-KO mice had significantly diminished CRC than controls (Fig. 3i), thus supporting increased capacity to Ca^2+^-induced PTP opening in IF1-KO mice. We included the same analysis in liver mitochondria of both genotypes to show no changes in CRC and the regulation of CRC by cyclosporine A (CsA) [42] as control of CRC studies (Supplementary Fig. S3i).

Interestingly, we observed that oligomycin, which inhibits both the ATP synthetic and hydrolytic activities of ATP synthase, significantly diminished ∆Ψm of IF1-ablated colonocytes with marginal effect in colonocytes of control mice (Fig. 3g), suggesting that the hydrolase activity of ATP synthase could be contributing to the generation of ∆Ψm in IF1-KO mitochondria. This idea was also supported by the increased content of ADP and AMP in IF1-KO mitochondria (Fig. 3f).

### IF1 ablation activates purine metabolism

**Fig. 4:**
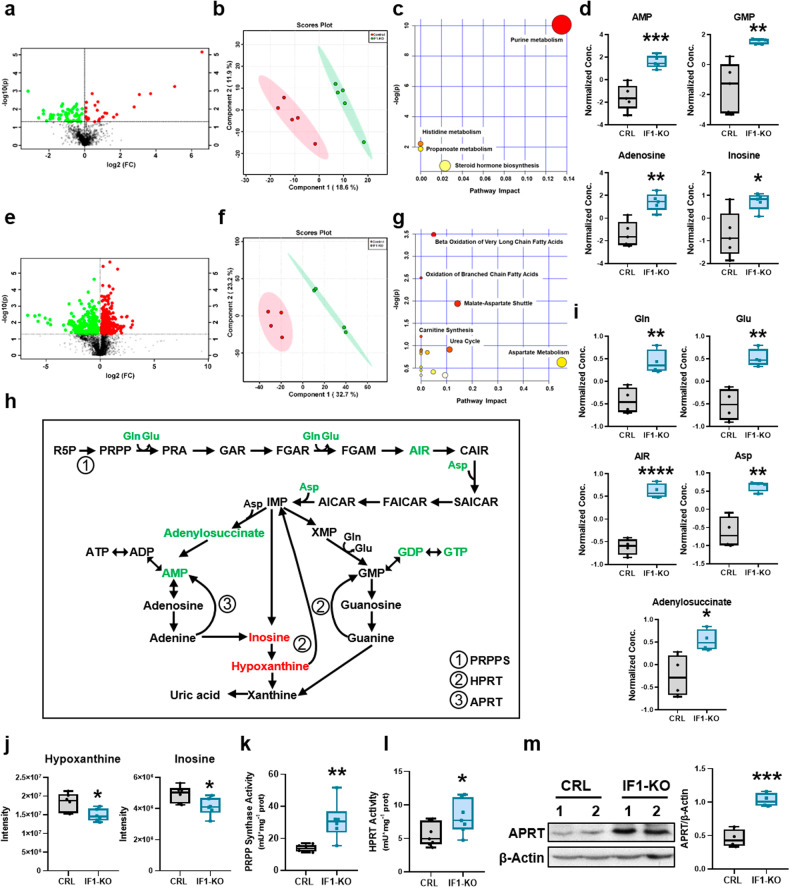
IF1 ablation induces de novo purine and salvage pathways. Untargeted metabolomic analysis of serum (**a**–**c**) and colon (**d**–**g**) of control (CRL) and IF1-KO mice. **a** Volcano plot identifying the metabolites that differ significantly in the serum between the two genotypes (*n* = 5). **b** Principal component analysis. **c** Pathway enrichment analysis of metabolites in the comparison IF1-KO vs. CRL mice. The node color is based on the *p*-values from integrated enrichment analysis, and the node radius represents the pathway impact values from topology analysis. **d** Box-and-whisker plots show purine metabolites levels in serum of IF1-KO and CRL mice (*n* = 5). **e** Volcano plot identifying the metabolites that differ significantly in the colon between the two genotypes (*n* = 4). **f** Principal component analysis. **g** Pathway enrichment analysis of metabolites in the comparison IF1-KO vs. CRL mice. **h** Scheme showing de novo purine and salvage pathways. Colon metabolites increased (green) and decreased (red) in IF1-KO mice are highlighted. **i**, **j** Box-and-whisker plots show metabolite levels in the colon of CRL and IF1-KO animals identified by untargeted (*n* = 4) (**i**) and targeted (*n* = 6) (**j**) analysis. Gln glutamine,Glu glutamic acid, Asp aspartic acid, AIR 5-aminoimidazole ribonucleotide. **k**, **l** Box-and-whisker plots showing the activity of phosphoribosyl pyrophosphate synthase (PRPP synthase) (*n* = 6) (**k**) and hypoxanthine-guanine phosphoribosyl transferase (HPRT) (*n* = 7) (**l**) in the colon of CRL and IF1-KO mice. **m** Representative Western blot of the expression of adenine phosphoribosyl transferase (APRT) in the colon of CRL and IF1-KO mice (*n* = 4). β-Actin is shown as loading control. Box-and-whisker plot shows APRT expression relative to β-Actin. *p ≤ 0.05, **p ≤ 0.01, ***p ≤ 0.001 when compared by Student’s *t*-test. See also Supplementary Fig. S4.

Untargeted metabolomic analysis of the serum of IF1-KO and control mice revealed significant differences between the two genotypes (Fig. 4a, b) and highlighted the activation of purine metabolism as distinguishing pathway between them (Fig. 4c). IF1-KO mice showed an increased concentration of serum AMP, GMP, adenosine and inosine (Fig. 4d). Moreover, IF1-KO mice showed diminished content of long-chain acyl-carnitines in serum (Supplementary Fig. S4a).

A similar metabolomic approach was carried out in colon extracts (Fig. 4e). PCA of the metabolites distinguishes both genotypes (Fig. 4f) and emphasized that β-oxidation of very long-chain fatty acids and branched-chain amino acids are two main pathways differentially affected (Fig. 4g), in agreement with the increased expression of the peroxisomal enzymes involved in β-oxidation (Supplementary Fig. S1d), the higher content of carnitine and 3-hydroxyhexadecanoylcarnitine in the colon (Supplementary Fig. S4b) and the diminished availability of serum long-chain acyl-carnitines (Supplementary Fig. S4a).

Remarkably, metabolomic analysis of the colon also identified an increased content of precursors of de novo purine synthesis pathway (Fig. 4h), such as 5-aminoimidazole ribonucleotide (AIR), adenylosuccinate and the amino acids glutamine, glutamic and aspartic acid in IF1-KO mice (Fig. 4i). Moreover, it should be mentioned that the colon of IF1-KO mice showed an increased content of acetylcholine (Supplementary Fig. S4c), a neurotransmitter that promotes the activation of Ca^2+^-dependent muscarinic receptors in gastrointestinal and muscle cells.

A targeted metabolomic approach confirmed that the purine nucleotides AMP, GDP and GTP were significantly increased in the colon of the IF1-KO mice (Supplementary Fig. S4d). Moreover, inosine and hypoxanthine, which are products of the catabolism of purine nucleotides (Fig. 4h), were significantly diminished (Fig. 4j), suggesting that they might be being reutilized in the purine nucleotide salvage pathway (Fig. 4h). In agreement with the activation of purine metabolism, we observed a sharp increase in the activity of phosphoribosyl pyrophosphate synthetase (PRPPS) (Fig. 4k) and of hypoxanthine-guanine phosphoribosyltransferase (HPRT) (Fig. 4l), enzymes of de novo purine synthesis and salvage pathways, respectively. Likewise, the expression of adenine phosphoribosyltransferase (APRT), which is involved in the adenine salvage pathway (Fig. 4h), was also significantly augmented in the colon of IF1-KO mice (Fig. 4m). Altogether, confirming the activation of both de novo and salvage purine pathways in the colon of IF1-KO mice.

Administration of a pulse of N^15^-glutamine into mice to assess in vivo the activity of de novo purine biosynthesis revealed that the incorporation of the tracer into 2xN^15^ and 3xN^15^-guanine was significantly augmented in IF1-KO mice when compared to controls (Supplementary Fig. S4e), further supporting that ablation of IF1 in the colon resulted in an enhanced de novo purine biosynthesis in these animals.

### Uncontrolled ATP synthase boosts cellular adenosine production

**Fig. 5:**
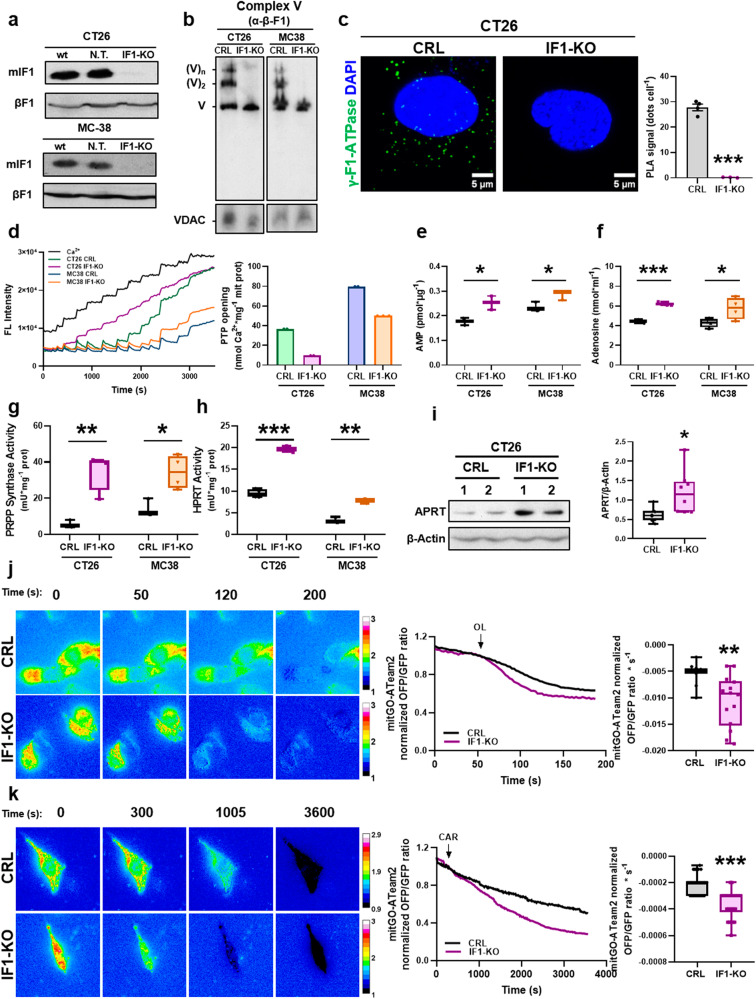
Uncontrolled ATP hydrolysis in mitochondria of IF1 knockout cells produces adenosine. IF1 knockout murine colon carcinoma cell lines CT26 and MC38 were developed. **a** Representative blots of IF1 expression in wild type (wt) and cells co-transfected with Cas9 and non-targeted sgRNA (N. T.) or guided to the *Atp5if1* gene (IF1-KO). β-F1-ATPase is used as loading control. **b** BN-PAGE of Complex V in mitochondria from CRL and IF1-KO CT26 and MC38 cells. β-F1 (Complex V) blots are shown. VDAC is shown as loading control. **c** Representative images of Proximity Ligation Assay (PLA) using γ-F1-ATPase as target (green dots). DAPI (blue) stained nuclei. Upper histograms show the number of PLA signals per cell in CRL (*n* = 4) and IF1-KO (*n* = 3) CT26 cells. **d** Calcium retention capacity (CRC) in mitochondria of CT26 and MC38 CRL (*n* = 2) and IF1-KO (*n* = 2–3) cells. Left, representative traces of the CRC. Right, histograms show the mean ± SEM of the amount of Ca^2+^ necessary to induce PTP opening. **e** Box-and-whisker plots show cellular levels of AMP in CT26 and MC38 CRL and IF1-KO cells (*n* = 3). **f** Box-and-whisker plots show the levels of adenosine released into the culture medium in CT26 and MC38 CRL and IF1-KO cells (*n* = 4). **g**, **h** Box-and-whisker plots show the activity of phosphoribosyl pyrophosphate synthase (PRPP synthase) (**g**) and hypoxanthine-guanine phosphoribosyl transferase (HPRT) (**h**) in CT26 and MC38 CRL and IF1-KO cells (*n* = 3-4). **i** Representative blot showing the expression of adenine phosphoribosyl transferase (APRT) in two independent preparations of CT26 CRL and IF1-KO cells. The box-and-whisker plot shows the corresponding quantitation (*n* = 4). β-Actin is shown as loading control. **j**, **k** Representative images and traces of mitochondrial ATP levels measured by the mitGO-ATeam2 probe at different times before and after the addition of oligomycin (OL) at 50 s (*n* = 11–14 cells per genotype assayed in three independent days) (**j**) or carboxyatractyloside (CAR) at 300 s (*n* = 27–36 cells per genotype assayed in three independent days) (**k**) in CT26 CRL and IF1-KO cells. Box-and-whisker plots show the rates of mitochondrial ATP turnover due to OL (**i**) or CAR (**j**) addition. *p ≤ 0.05, **p ≤ 0.01, ***p ≤ 0.001 when compared by Student’s *t*-test. See also Supplementary Fig. S5.

To verify the implication of the ATP synthase in the activation of purine metabolism, we developed the IF1-ablated murine colon carcinoma cell lines CT26 and MC38 (Fig. 5a). Consistent with in vivo data, depletion of IF1 resulted in increased ATP synthetic and hydrolytic activities of ATP synthase in both cell lines (Supplementary Fig. S5a). Moreover, the lack of IF1 vanished the oligomeric assemblies of ATP synthase as assessed in BN-gels (Fig. 5b) and by Proximity Ligation Assays (PLA) (Fig. 5c) using the γ-subunit of ATP synthase as the target of the primers of DNA polymerase. Likewise, lack of IF1 increased the rates of respiration in the CT26 cell line using palmitate as substrate (Supplementary Fig. S5b), the latter in agreement with the activation of β-oxidation in IF1-KO mice (Fig. 2f and Supplementary Fig. S1d). In contrast to the data in the animal model, ∆Ψm was lower in IF1-KO cells when compared to controls and not affected by oligomycin treatment (Supplementary Fig. S5c). Interestingly, although we observed no relevant differences in the intramitochondrial Ca^2+^ content in both CT26 and MC38 cell lines (Supplementary Fig. S5d), the CRC was significantly diminished in both IF1-KO cell lines when compared to controls (Fig. 5d).

The cellular content of ATP and ADP was not affected by the ablation of IF1 in the cell lines (Supplementary Fig. S5e). However, a significant increase in cellular AMP concentrations was observed (Fig. 5e), suggesting that it might be generated by the uncontrolled hydrolase activity of ATP synthase. In line with this idea, both cell lines overproduced and released adenosine to the culture medium (Fig. 5f). Consistent with the activation of purine de novo and salvage pathways observed in the colon of IF1-KO mice, both the activities of PRPPS (Fig. 5g) and HPRT (Fig. 5h) were significantly augmented in CT26 and MC38 cell lines. Moreover, APRT expression was also increased in IF1-KO CT26 cells (Fig. 5i) but not in MC38 cells (Supplementary Fig. S5f), perhaps because this cell line displayed the lowest activity of ATP synthase (Supplementary Fig. S5a). Overall, the results in cell lines support that ablation of IF1 is responsible for the activation and depolymerization of ATP synthase resulting in enhanced purine metabolism and the overproduction of extracellular adenosine.

### Uncontrolled ATP synthase enhances mitochondrial ATP turnover

To confirm an enhanced rate of ATP hydrolysis in the mitochondria of IF1-ablated mice, we studied the initial rates of ATP disappearance using a FRET-ATP sensitive mitGO-ATeam2 probe in CT26 cells [43]. When cells were treated with oligomycin (OL) to inhibit ATP synthase/hydrolase activities, the intramitochondrial ATP degradation rate was higher in IF1-KO CT26 cells when compared to controls (Fig. 5j). Similar findings, although of lesser intensity, were obtained when the import of ADP into mitochondria was inhibited with carboxyatractyloside (CAR) (Fig. 5k). Altogether, supporting that the activation of purine metabolism in the colon and the subsequent accumulation of adenosine in serum of IF1-KO mice results from an uncontrolled ATP hydrolytic activity of ATP synthase in intestinal mitochondria.

### IF1 ablation affects gastrointestinal homeostasis

**Fig. 6:**
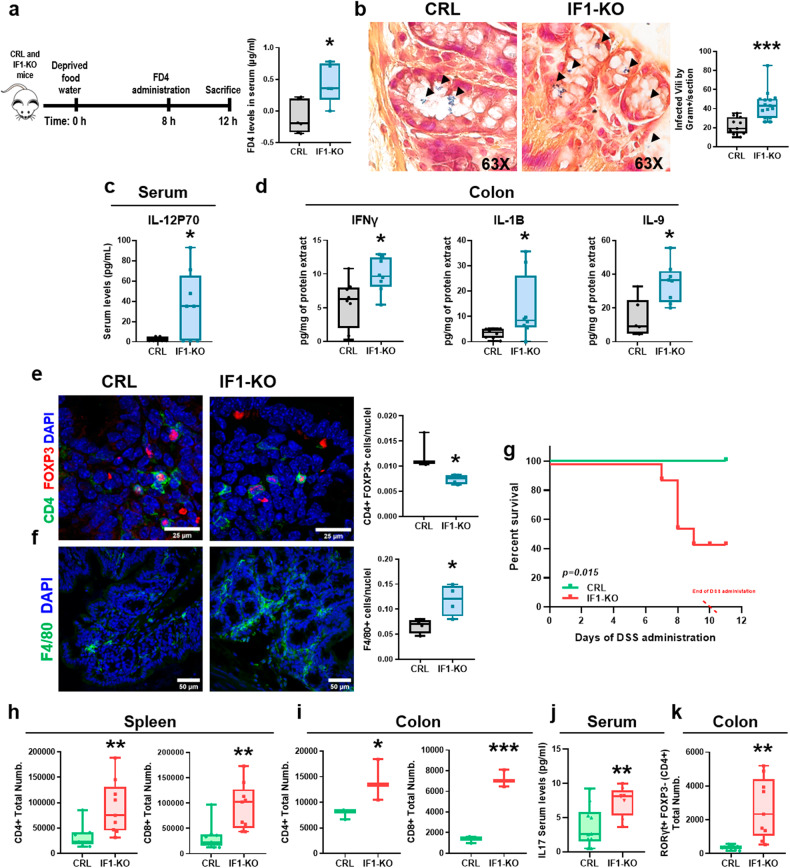
The lack of IF1 promotes a pro-inflammatory phenotype and damages the permeability of the intestinal barrier. **a** Analysis of the permeability of the intestinal barrier using the fluorescent dextran (FD4). The box-and-whisker plot shows the serum levels of FD4 in CRL (*n* = 6) and IF1-KO (*n* = 5) mice. **b** Left, representative images of the histological analysis of the infiltration of Gram+ bacteria in the colon of CRL (*n* = 4) and IF1-KO (*n* = 5) mice. Right, box-and-whisker plot shows the number of villi infected by Gram+ bacteria. **c**, **d** Multiplexed quantitative assay of cytokines and chemokines. Box-and-whisker plots show cytokine levels in the serum (**c**) and colon (**d**) of CRL and IF1-KO mice (*n* = 8). **e**, **f** Representative immunofluorescence images showing T regulatory subset (CD4^+^, green, FOXP3^+^, red) (**e**) and total macrophages (F4/80^+^, green) (**f**) in the colon of CRL (*n* = 3-4) and IF1-KO (*n* = 4) mice. Box-and-whisker plots show the corresponding quantifications. DAPI (blue) stained nuclei. **g** Kaplan–Meier survival analysis of CRL (*n* = 8) and IF1-KO (*n* = 9) mice after treatment with 2% dextran sodium sulfate (DSS) in the drinking water. The *p*-value of the log-rank test is shown. **h**, **i** Box-and-whisker plots show the total number of CD4^+^ (CD4^+^) and CD8^+^ (CD8^+^) lymphocytes in the spleen (*n* = 3) (**h**) and colon (*n* = 3) (**i**) of CRL and IF1-KO mice after 6 days of treatment with 2% DSS. **j** Box-and-whisker plot shows the serum levels of cytokine IL-17 in CRL (*n* = 10) and IF1-KO mice (*n* = 8) after 10 days of treatment with 2% DSS. **k** Box-and-whisker plots show the total number of Th17 lymphocytes (RORγt^+^ FOXP3^-^ CD4^+^) in the colon of CRL and IF1-KO mice (*n* = 3) after 6 days of treatment with 2% DSS. *p ≤ 0.05, **p ≤ 0.01, ***p ≤ 0.01 when compared by Student’s *t*-test. See also Supplementary Figs. S6 and S7.

To understand the biological relevance of IF1 in the intestine, a quantitative proteomic analysis was developed; 24,491 peptides were identified corresponding to 88 differentially expressed proteins (Supplementary Fig. S6a–c) that discriminated the two genotypes (Supplementary Fig. S6d). Ingenuity Pathway Analysis (IPA) indicated that inflammation, tissue development and gastrointestinal disease (colitis) were activated in IF1-KO mice (Supplementary Fig. S6e). In this regard, the determination of the integrity of the intestinal barrier by assessing its permeability to a bolus of fluorescent FD4 dextran confirmed that IF1-KO mice have a permeable intestinal barrier (Fig. 6a). Consequently, IF1-KO mice also showed an enhanced infiltration of Gram+ bacteria in intestinal villi (Fig. 6b). However, we observed no changes in gene expression analysis of relevant proteins involved in the zonula adherens/occludens, tight junctions and desmosomes between the two genotypes (Supplementary Fig. S7a). Western blotting (Supplementary Fig. S7b) and immunofluorescence (Supplementary Fig. S7c) of E-cadherin confirmed the absence of differences between the two genotypes.

### IF1-KO mice have an altered immune response and compromised survival upon inflammation

In agreement with a predicted activation of the inflammatory response in IF1-KO mice (Supplementary Fig. S6e), we observed that levels of interleukin IL-12P70, which is produced by macrophages and dendritic cells in response to bacterial infection, were significantly augmented in the serum of IF1-KO mice (Fig. 6c). These findings are in accordance with the higher permeability and penetration of bacteria in their intestine (Fig. 6a, b). IL-12 links both the innate and adaptive immune responses of the tissue by triggering the activation of Natural Killer (NK) cells and the differentiation of naïve CD4+ T cells into Th1 subsets. Moreover, IL-12 also activates cytotoxic CD8+ T cells to combat infection. Indeed, the colon of IF1-KO mice showed increased content of the pro-inflammatory cytokines IFNγ, IL-1B and IL-9 (Fig. 6d). Consistent with this pro-inflammatory phenotype, we observed a significant reduction in T regulatory cells (Tregs) (Fig. 6e) and an increased content of macrophages (Fig. 6f) in the colon of IF1-KO mice. Interestingly, and despite the pro-inflammatory phenotype of IF1-KO mice, their lifespan was not significantly compromised when compared to controls (Supplementary Fig. S7d).

However, upon the administration of a standard pro-inflammatory-DSS protocol, 60% of IF1-KO mice died before completing the study (Fig. 6g). As expected, DSS administration promoted in IF1-KO mice a sharper decrease in body weight (Supplementary Fig. S7e) and in colon length (Supplementary Fig. S7f) and the upregulation of CD4^+^ and CD8^+^ lymphocytes in the spleen (Fig. 6h) and colon (Fig. 6i), in agreement with their higher inflammatory stress response. Moreover, serum IL-17 levels (Fig. 6j) and colon Th17 lymphocytes (RORγt^+^FOXP3^-^) (Fig. 6k) were significantly increased in IF1-ablated mice.

Altogether supporting that their altered barrier permeability backs a stronger bacterial infection and septic-induced death. Overall, ablation of IF1 promotes an altered mitochondrial function that affects the intestinal epithelial barrier fomenting bacterial infection and an aggravated pro-inflammatory response.

### Adenosine hampers the immune response of the intestine

**Fig. 7:**
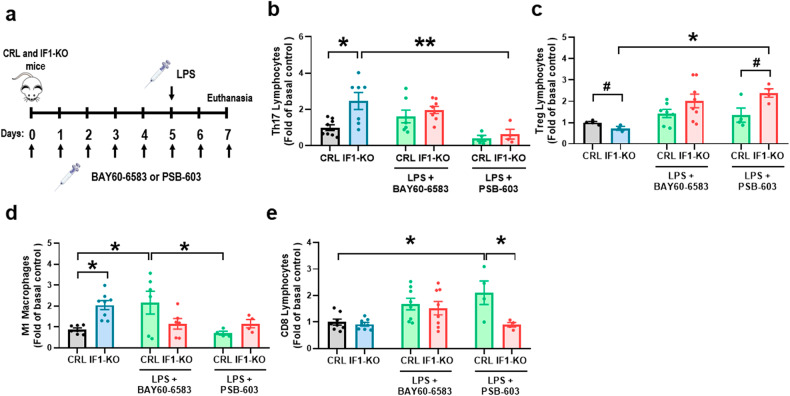
Adenosine, through ADORA2B receptors, stimulates an autoimmune phenotype. **a** Control (CRL) and IF1-KO mice were non-treated or treated with the ADORA2B agonist BAY60-6583 (daily dose of 1 mg kg^−1^) or antagonist PSB-603 (daily dose of 5 mg kg^−1^) for 7 days. On the fifth day, lipopolysaccharide (LPS) was injected (single dose of 8 mg kg^−1^), and the animals were sacrificed 2 days later for the analysis of immune cell subsets. **b**–**e** Histograms show the percentage (mean ± SEM) of Th17 (CD4^+^ IL17A^+^) (**b**), Treg (CD4^+^ FOXP3^+^) (**c**), M1 macrophages (CD45^+^ CD11b^+^ F4/80^low^ Ly-6C^high^) (**d**) and CD8^+^ lymphocytes (CD45^+^ CD8^+^ DAPI^−^) (**e**) in the colon of CRL and IF1-KO mice non-treated (*n* = 8) or treated with LPS and the agonist BAY60-6583 (*n* = 6–8) or antagonist PSB-603 (*n* = 4). #*p* ≤ 0.05 when compared according to the Student’s *t*-test. *p ≤ 0.05, **p ≤ 0.01 when compared according to the one-way ANOVA test and the Tukey multiple correction test. See also Supplementary Fig. S7.

The immunometabolite adenosine, which accumulates in the serum of IF1-KO mice (Fig. 4d) and in the medium of IF1-KO cells (Fig. 5f), has recently been shown to have pro-inflammatory effects when it acts on ADORA2B receptors [44, 45]. To investigate the implications of adenosine in the immune response of the colon, mice were treated with the selective ADORA2B agonist (BAY-60-6583) or antagonist (PSB-603) under LPS-induced inflammatory conditions (Fig. 7a). Analysis of total colon content of CD4 lymphocytes (CD4^+^) (Supplementary Fig. S7g), Th17 (CD4^+^IL17A^+)^ (Fig. 7b) and Treg (CD4^+^FOXP3^+^) (Fig. 7c) subsets, as well as M1 macrophages (CD11b^+^) (Fig. 7d) and neutrophils (CD11b^+^Ly-6G^+^) (Supplementary Fig. S7h) confirmed the pro-inflammatory phenotype of IF1-KO mice under basal conditions. No relevant differences were observed in the content of the Th1 subset (CD4^+^IFNγ^+^) (Supplementary Fig. S7i) and in CD8 lymphocytes (CD8^+^) (Fig. 7e). Remarkably, upon inflammation, the treatment with the agonist did not affect the colon content of Th17 (Fig. 7b), Treg (Fig. 7c) and CD8 lymphocytes (Fig. 7e), whereas it increased the content of M1 macrophages (Fig. 7d). In contrast, upon inflammation, the treatment with the ADORA2B antagonist significantly reduced the content of Th17 subset (Fig. 7b), CD8 lymphocytes (Fig. 7e) and M1 macrophages (Fig. 7d) and significantly increased the Treg population in the colon (Fig. 7c). Altogether these results support that adenosine, produced through activation of futile ATP hydrolysis in mitochondria, acts via ADORA2B receptors to promote the pro-inflammatory phenotype of IF1-KO mice.

## Discussion

In agreement with previous findings in in vitro cellular models [23, 46, 47], pharmacologic in vivo approaches [25, 46], genetic mouse models of loss and gain of function of IF1 [15–18, 20] and recent cryo-EM studies [13, 14], we support that IF1 binds and inhibits a fraction of mitochondrial ATP synthase in vivo. In other words, mitochondria from IF1-expressing tissues contain both active and IF1-inactive ATP synthase under phosphorylating conditions.

cAMP-dependent activation of PKA promotes IF1 phosphorylation at S39, which prevents its binding to ATP synthase [46]. Remarkably, recent findings have demonstrated that the binding of kynurenic acid to the GPR35 orphan receptor prevents the phosphorylation of IF1, providing an anti-ischemic mechanism for ATP conservation in human and mouse cardiomyocytes [25, 48]. Wyant et al. showed that dephosphorylated IF1 binds to ATP synthase and promotes enzyme oligomerization [25], in agreement with recent cryo-EM structures of the oligomeric inhibited enzyme [13, 14, 20]. Interestingly, colon extracts have both phospho- and dephospho-IF1 under normal physiological conditions [12], thus supporting that the content of dephospho-IF1 controls the fraction of oligomeric and inhibited ATP synthase in the colon.

We show that genetic ablation of IF1, which is a highly abundant mitochondrial protein in the intestinal epithelium [12], promotes a profound alteration of mitochondrial structure and function, resulting in an altered immune response of the tissue that impairs the permeability of the intestinal barrier. Mechanistically, *knockout* of IF1 in colonocytes promotes a large increase in ATP hydrolytic and synthetic activities of ATP synthase and installs a futile cycle of mitochondrial hydrolysis of ATP, as shown in FRET imaging experiments. Futile ATP hydrolysis stimulates the accumulation of adenosine in the serum of IF1-KO mice and in the culture medium of cells and triggers the activation of de novo and salvage pathways of purine metabolism, most likely to compensate for the lower energy state [49, 50]. Adenosine, acting through ADORA2B purinergic receptors, induces a Th17/Treg-type pro-inflammatory immune response [51] in the colon that affects the permeability of the intestinal barrier compromising IF1-KO mice survival under inflammatory conditions. Overall, the model of IF1 loss-of-function in the intestinal epithelium highlights the relevance of the mitochondrial IF1/ATP synthase axis in tissue immune response.

We show that a consequence of the content of IF1 in mitochondria is the production of ATP synthase oligomers supporting an active role for IF1 in enzyme oligomerization in vivo in intestinal cells, in agreement with previous findings [13, 14, 16, 18, 20]. Oligomers of ATP synthase are required for the generation of mitochondrial cristae [2]. Hence, we observed that ablation of IF1 also has a profound effect on mitochondrial structure and cristae length. However, the alteration of mitochondrial structure in IF1-ablated mice cannot be solely ascribed to a deficit of ATP synthase oligomerization because the expression of components of the MICOS complex, which are also involved in building cristae structured [52], are also diminished by deletion of IF1. Moreover, the ablation of IF1 specifically minimized the expression and activity of mitochondrial respiratory complexes without affecting other membrane proteins of mitochondria. These might be a consequence of cristae disorganization [53], although the precise mechanisms involved in the specific repression of the expression of components of the respiratory chain in vivo remain to be investigated.

Mitochondria of IF1-ablated mice showed large intramitochondrial Ca^2+^ concentrations, Ca-P deposits, increased Ca^2+^ efflux rate and a diminished CRC when compared to mitochondria in controls, supporting a relevant role for IF1 in normal mitochondrial function in the intestinal epithelium. Interestingly, the alteration in CRC is also observed in IF1-ablated cell lines. However, that is not the case for the intramitochondrial calcium concentration and the expression of components and activity of the respiratory chain. These results highlight the differences between the in vivo and in vitro systems, most likely because the former integrates the activity of different players of the biological response. Indeed, in vivo findings indicated that the neurotransmitter acetylcholine was significantly increased in the colon of the IF1-KO mice (Supplementary Fig. S4c). Cholinergic signaling by acetylcholine plays a fundamental role in regulating intestinal function since it stimulates smooth muscle contraction and intestinal secretions [54, 55]. The main sources of acetylcholine in the intestine include cholinergic neurons and acetylcholine-producing immune cells [56, 57]. Activation of muscarinic receptors of intestinal epithelial cells by acetylcholine increases the cellular Ca^2+^ content [58, 59]. Hence, we suggest that the increased Ca^2+^ accumulation observed in the mitochondria of IF1-KO mice might be promoted by enhanced cholinergic signaling.

The driving force for Ca^2+^ accumulation in mitochondria is ΔΨm [40]. Consistently, calcium accumulation is only observed in the mitochondria of colonocytes of IF1-KO mice, paralleling the differences in ΔΨm observed. Remarkably, a large fraction of ΔΨm in IF1-KO colonocytes was partially collapsed by oligomycin, supporting that a fraction of ΔΨm is dependent on the reverse function of the ATP synthase. Although the reverse functioning of ATP synthase is also operative in the IF1-KO cell lines, its contribution to ΔΨm is negligible, perhaps because their electron transport chain is not compromised, as shown by the colonocytes in vivo. The formation of Ca-P deposits is assumed to be a mitochondrial response to drop the threshold of PTP opening [39, 40, 60]. Indeed, mitochondria of IF1-KO colonocytes and IF1-KO cell lines do have a much lower CRC than their respective controls, indicating their higher susceptibility to PTP opening. This may underlie the increased cell death observed in colonocytes of IF1-KO mice and thereby could also contribute to compromise barrier function of the intestinal epithelium. Recently, the participation of the ATP synthase in PTP formation has been quite well delineated [5, 6, 8, 14]. It is probable that the loss of ATP synthase oligomers due to IF1 ablation increases the susceptibility of PTP opening. In this regard, it is likely that monomeric ATP synthase is more efficient in transducing the Ca^2+^-induced conformational changes in the F1 domain to promote the opening of the PTP than the IF1-bound tetrameric enzyme [14, 48, 61]. In fact, the overexpression of IF1 is known to prevent cell death [7, 29, 62]. This would explain why IF1-ablated cell lines that do not show differences in intramitochondrial calcium concentration display lower CRC.

Inflammatory bowel diseases (IBD) exhibit loss of intestinal barrier integrity and aberrant immune cell responses [63]. Mitochondrial dysfunction is a key element in the loss of intestinal homeostasis as well as in the pathophysiology of IBD [64, 65]. In fact, IBD patients show reduced levels of gut ATP [66, 67] and their enterocytes present mitochondria with irregular cristae [67–69]. Experimental mouse models with induced colitis also show altered mitochondrial structure [70], supporting that bioenergetic dysfunction of mitochondria is involved in IBD. In this same line, our mouse model shows that the activation of futile ATP hydrolysis by ATP synthase results in the accumulation of the immunometabolite adenosine. Although adenosine is widely known to have anti-inflammatory effects [71], recent findings support that when it acts on purinergic adenosine A2B (ADORA2B) receptors [45], it has a pro-inflammatory role [44]. Herein, we propose that the accumulated adenosine, acting through ADORA2B receptors, promotes a pro-inflammatory phenotype in the colon of the IF1-KO mice. This autoimmune response is evidenced by an increase in colon infiltrating Th17 cells that contribute to the alteration of the permeability of the intestinal barrier, in agreement with recent suggestions [72]. Moreover, the phenotype is aggravated by the decreased colon infiltration of Treg cells, which are known to increase self-tolerance [73], and hence it is compatible with an autoimmune scenario, which is partially reverted when mice are treated with an ADORA2B antagonist.

Overall, the results highlight the essential role that IF1 plays in the intestinal epithelium to prevent wasteful hydrolysis of ATP under physiological conditions and further provide a genetic mouse model of IBD that stresses the role of IF1/ATP synthase axis as the link between mitochondria and the tissue immune response.

## Materials and methods

### Genetically engineered mice

Mouse experiments were carried out after approval of the institutional review board (Ethical Committee of the UAM, CEI-101-1891-A325) in compliance with animal policies and ethical guidelines of the European Community. Mice were housed in the Animal Facility of the CBMSO with a 12-h light/12-h dark cycle and temperatures of 18–23 °C with 40–60% humidity. C57BL/6NTac-Atpif1^tm1a(EUCOMM)Wtsi/WtsiCnbc^ mouse line [74] was acquired from The European Mouse Mutant Archive (EMMA, RRID: IMSR_EM:05233) to develop IF1-floxed mice by breeding it with B6;SJL-Tg(ACTFLPe)9205Dym/J mice (The Jackson Laboratory, RRID: IMSR_JAX:003800) [75]. IF1-floxed mice [18] were bred with Villin-CreERT2:Tg(Vil-cre/ERT2)23Syr mouse line (The Jackson Laboratory, J:92295) [36], a tamoxifen-inducible mice which expresses the Cre-recombinase fused with the estrogen receptor (ER^T2^) under the control of villin promoter in order to obtain the inducible IF1 *knockout* mice. The administration of tamoxifen in chow (Envigo, TD.55125) results in the activation of Cre-recombinase and, thus, in the generation of the IF1-KO mice in the gut epithelium. Mice were maintained on C57BL/6J background. Experiments were carried out with 2- to 4-month-old IF1-KO male mice using the villin-Cre-ER^T2^ male mice as control animals. Tamoxifen was administered in two cycles of 5 days per week to 2- to 4-month-old mice until the day of sacrifice.

In order to minimize the number of animals, we used power analysis to calculate the minimum sample size using the free software DOEUMH (https://samplesizeumh.shinyapps.io/DOEUMH) based on the Trial Size library of the R program (R Core Team). We selected the procedure Means—ANOVA, fixing the significance to 0.05, power to 0.08 and a drop-out of 5%. We took into consideration differences between averages of about 1.5- to 2-fold. Minimum number of mice/group: 3–4 mice/group. Randomization was assessed by equally distributing experimental groups across multiple cages and balancing the location of the mouse cages on the racks. Tests were performed in a non-blinded fashion.

### Cell lines and culture conditions

Cells were cultured in a humidified incubator at 37 °C with a controlled atmosphere of ambient air 10% CO_2_. Murine colorectal carcinoma cell line CT26 (ATCC, CRL-2638) or IF1-KO CT26 were grown in RPMI media supplemented with 10% fetal bovine serum (FBS, MilliporeSigma, F7524). Murine colorectal carcinoma cell line MC38 (Kerafast, ENH204-FP) or IF1-KO MC38 were grown in DMEM media supplemented with 10% fetal bovine serum (FBS, MilliporeSigma, F7524). The generation of stable IF1-KO cells was carried out by the CRISPR-Cas9 technique [12]. Cells were co-transfected with two plasmids (MLM3636-sgRNA-mIF1-1 and MLM3636-sgRNA-mIF1-2) that express two guides for *Atp5if1* and a plasmid that contains the nuclease Cas9 and GFP (pSpCas9(BB)-2A-GFP, PX458) (Addgene, RRID: Addgene_48138). Transfection was carried out using Lipofectamine 3000 (Thermo Fisher, L3000015) following the manufacturer’s instructions. After 48 h, GFP^+^ cells were sorted in FACSAria Fusion equipment (BD Biosciences) from the Cytometry Facility (CBMSO, Spain), and they were expanded for future experiments. Cells transfected with a plasmid that express a scramble guide and the plasmid that contains the nuclease Cas9 and GFP were used as controls. The *Atp5if1* CRISPR-Cas9 mediated gene knockout was checked by Western blot.

### PCR genotyping

The primer sequences for genotyping any *Atp5if1* allele (wild-type or *floxed*) and Cre recombinase are listed in Supplementary Table S1.

### Isolation of mitochondria

Mitochondria were isolated from fresh tissue, which was minced and homogenized in a glass-glass homogenizer with 7 ml mg^−1^ of cold buffer A (320 mM sucrose, 1 mM EDTA, 10 mM Tris-HCl, pH 7.4). Nuclei and unbroken cells were removed by centrifugation at 800×*g* for 10 min at 4 °C, and mitochondria were pelleted by centrifugation at 7500×*g* [16].

### Determination of ATP synthase and hydrolase activities

Fresh mitochondria or digitonin-permeabilized cells were used for determining mitochondrial ATP production [76]. Samples were resuspended in respiration buffer (225 mM sucrose, 10 mM KCl, 5 mM MgCl_2_, 0.05 % BSA, 10 mM potassium-phosphate buffer, 1 mM EGTA and 10 mM Tris-HCl, pH 7.4) supplemented with EDTA-free protease inhibitor cocktail (MilliporeSigma, 11836170001) and phosphatase inhibitor cocktail-2 (MilliporeSigma, P5726) and added to a luminometer plate reader. ATP production was measured as luminescence production in respiration buffer containing 0.1 mM ADP, 5 mM succinate, 0.15 µM P^1^,P^5^-di(adenosine-5′) pentaphosphate, 0.165 mg ml^−1^ D-luciferin (Invitrogen, L2916) and 0.003 mg ml^−1^ luciferase (Merck KGaA, SRE-0045). Relative light units were converted into ATP concentration using an ATP standard curve.

Isolated mitochondria from the colon or cells were used for the spectrophotometrical determination of ATP synthase hydrolytic activity by an assay coupled to pyruvate kinase and lactate dehydrogenase, following the changes in absorbance at 340 nm (A_340_) [76]. Then, 50 μg protein of isolated mitochondria were resuspended in 20 μl of reaction buffer (50 mM Tris-HCl, pH 8.0; 5 mg ml^−1^ BSA, 20 mM MgCl_2_, 50 mM KCl) supplemented with EDTA-free protease inhibitor cocktail (MilliporeSigma, 11836170001) and phosphatase inhibitor cocktail-2 (MilliporeSigma, P5726). The ATP hydrolysis was determined in a luminometer in a completed reaction buffer with 5 μM FCCP, 1 μM antimycin A, 10 μM PEP, 2.5 mM ATP, 1 mM NADH, 4 units of LDH and 4 units of PK in a final volume of 100 µl per well.

Inhibition of the ATP synthase and hydrolase activities was accomplished by the addition of 20 μM oligomycin.

### Mitochondrial enzyme activities

Isolated mitochondria from the intestine were used for the spectrophotometric determination of the activity of complexes I-IV [77]. Complex I activity was measured at A_340_ using 100 µg of mitochondria in 1 ml C1/C2 buffer (25 mM K_2_HPO_4_, 5 mM MgCl_2_, 3 mM KCN and 2.5 mg ml^−1^ BSA) containing 0.1 mM UQ1, 0.1 M NADH and 1 mg ml^−1^ antimycin A. Inhibition of the activity was accomplished by the addition of 1 μM rotenone. Complex II activity was measured at A_600_ using 100 µg of mitochondria in 1 ml C1/C2 buffer containing 30 µM DCPIP, 1 µM rotenone, 1 µM antimycin A, 10 mM succinate and 6 mM phenazine methosulfate. Complex III activity was assessed by the Mitochondrial Complex III Activity Assay Kit (MilliporeSigma, MAK360-1KT) following the manufacturer’s instructions. Complex IV was measured at A_550_ using 100 µg of mitochondria in 10 mM KH_2_PO_4_, pH 6.5, 0.25 M sucrose and 1 mg ml^−1^ BSA containing 10 µM reduced cytochrome c. Cytochrome c solution was freshly reduced by adding some crystals of sodium dithionite. Inhibition of the activity was accomplished by the addition of 240 µM KCN.

### Protein extraction and Western blot analysis

Tissues, cells or isolated mitochondria were homogenized in Tissue Protein Extraction Reagent (T-PER) (Thermo Fisher, 78510) supplemented with EDTA-free protease inhibitor cocktail (MilliporeSigma, 11836170001) and phosphatase inhibitor cocktail-2 (MilliporeSigma, P5726). Homogenates were freeze-thawed three times in liquid nitrogen and clarified by centrifugation at 11,000×*g* for 30 min at 4 °C. Protein concentration was determined with Bradford reagent (Bio-Rad Protein Assay, 5000001). Protein extracts (30 µg) from the colon, cell lysates or isolated mitochondria were fractionated on SDS-6%, -9% or -12% PAGE and transferred onto PVDF (0.45 μm pore, Immobilon-P, Merck, IPVH00010) or nitrocellulose (GE Healthcare Life Sciences, 15289804) membranes. Membranes were blocked with 5% nonfat dried milk in Tris-buffered saline (TBS) with 1% Tween 20 for 1 h at room temperature and incubated with the primary antibody diluted in 3% BSA and 0.05% NaN_3_ in TBS overnight at 4 °C. The primary antibodies used are listed in the Supplementary Table S2. Peroxidase-conjugated anti-mouse or anti-rabbit IgGs (1:5,000) (Nordic Immunology) were diluted in TBS with 1% Tween 20 and used as secondary antibodies. The Novex® ECL (Thermo Fisher, WP20005) system was used to visualize the bands. The intensity of the bands was quantified using a GS-900^TM^ Calibrated Densitometer (Bio-Rad) and ImageJ Software (National Institutes of Health).

The carbonylation of proteins was detected with the OxyBlot Protein Oxidation Detection Kit (MilliporeSigma, S7150) following the manufacturer’s instructions.

### Blue-native (BN) and Clear-native (CN) gel electrophoresis

Mitochondrial pellets were suspended in 50 mM Tris-HCl pH 7.0 containing 1 M 6-aminohexanoic acid at a final concentration of 10 mg ml^−1^. The membranes were solubilized by the addition of 10% digitonin (4:1 digitonin:mitochondrial protein). For BN-PAGE, 5% Serva Blue G dye (Serva, 35050) in 1 M 6-aminohexanoic acid was added to the solubilized membranes. For CN-PAGE, the Serva Blue G dye was replaced by 0.1% Ponceau Red and 5.5% glycerol. In both BN and CN, Native PAGE™ Novex® 3–12% Bis-Tris Protein Gels (Life Technologies, BN1001BOX) were loaded with 50 μg of mitochondrial protein. The electrophoresis was performed at a constant voltage of 70 V for 15 min, followed by 1 h at a constant amperage of 10 mA. BN cathode buffer: 50 mM Tricine, 15 mM Bis-Tris, pH 7.0, 0.02 % Serva blue G; BN anode buffer: 50 mM Bis-Tris, pH 7.0; CN-cathode buffer: 50 mM Tricine, 15 mM Bis-Tris, 0.05 % sodium deoxycholate, pH 7.0, CN-anode buffer: 50 mM Bis-Tris, pH 7.0. For Clear-Native gels, after fractionation of mitochondrial proteins, the gels were incubated with 270 mM glycine, 35 mM Tris, 8 mM ATP, 14 mM MgSO_4_, 0.2 % Pb(NO_3_), pH 8.4 to assess the hydrolytic activity of the ATP synthase. For Blue-Native gels, after fractionation, the gels were electroblotted onto PVDF membranes. Membranes were further processed for immunoblotting. The primary antibodies are listed in the Supplementary Table S2.

### Proximity ligation assay (PLA)

Cells cultured on coverslips were fixed with 4 % paraformaldehyde (PFA), permeabilized with 0.1 % Triton X-100 and then Duolink^®^ PLA Probes and Fluorescent Detection Reagents were used following the manufacturer’s protocol. In brief, fixed and permeabilized CT26 cells were blocked with Duolink Blocking Solution and incubated sequentially with anti-γ-F1-ATPase antibody as primary antibody and anti-mouse PLUS and MINUS probes as secondary antibodies. Then, samples were incubated with 1x Ligation buffer with Ligase for 30 min at 37 °C, and next, with 1x Amplification buffer with Polymerase for 100 min at 37 °C. In situ mounting Medium with DAPI was used to mount the samples. Cellular fluorescence was analyzed by confocal microscopy in an A1R+ microscope (Nikon) at CBMSO Optical and Confocal Microscopy Facility and processed with ImageJ software.

### Co-immunoprecipitation assays

Co-immunoprecipitation was performed essentially as described [46]. Isolated mitochondria were lysed in 50 mM Tris-HCl, pH 6.0, 150 mM NaCl, 0.5% Nonidet P40, 0.5% Triton X-100, complete EDTA-free protease inhibitor cocktail (MilliporeSigma, 11836170001) and phosphatase inhibitor cocktail-2 (MilliporeSigma, P5726). Then, 20 µl of anti-IF1 antibody [12] were bound to EZ View Red Protein G Affinity Gel (MilliporeSigma, E3403) for 3 h at room temperature in RIPA buffer (0.1 M Tris-HCl, pH 8.0, 0.5 M NaCl, 2% Triton X-100, 1% sodium deoxycholate, 0.2% SDS). Then, protein from lysates (50 or 100 μg) was incubated with the anti-IF1 antibody bound to EZ View Red Protein G Affinity Gel overnight at 4 °C. β-F1 was detected with an anti-β-F1 antibody [78] (1:500) labeled with Cy5 using Amersham™ CyDye™ Reactive Dye Pack (GE Healthcare, PA25001) and IF1 was detected with rabbit anti-IF1 [12] (1:1000).

### Electron microscopy

Colon tissue sections were fixed with 4% PFA and 2% glutaraldehyde in 0.1 M phosphate buffer, pH 7.4 and samples were treated with 1% osmium tetroxide plus 0.8 % potassium ferricyanide in water at 4 °C for 1 h, dehydrated with ethanol, and embedded in TAAB 812 epoxy resin (TAAB Laboratories Equipment, E202). Sample preparation was carried out by the Electron Microscopy Facility (CBMSO, Spain). Ultrathin 80 nm sections of the embedded tissue were obtained using an Ultracult E ultramicrotome and mounted on carbon-coated copper slot grids. Sections were stained with uranyl acetate and lead citrate. Images were examined at 80Kv in a Jeol JEM1400 Flash Transmission Electron Microscope and a CMOS Oneview (4Kx4K) camera (Gatan). Mitochondrial shape descriptors and cristae measurements were obtained manually in ImageJ [79, 80].

### Determination of adenine nucleotides and adenosine

Isolated colon mitochondria were homogenized in 9 volumes of 100 mM Tris and 4 mM EDTA buffer, pH 7.75, preheated to 95 °C. Samples were incubated at 100 °C for 2 min and centrifuged at 1000×*g* for 2 min at 4 °C. The supernatant (50 µl) was used to determine ATP with 1 volume of reaction buffer. Luminescence was measured in 96-well plates with FLUOstar Omega (BMG Labtech) using ATP Bioluminescence Assay Kit HS II (Roche, 11699695001) following the manufacturer’s instructions.

Proteins from isolated colon mitochondrial were precipitated in 6 volumes of 6% perchloric acid and neutralized with KOH for the determination of ADP and AMP using ADP Assay Kit (MilliporeSigma, MAK133) or AMP Assay Kit (Abcam, ab273275), respectively, following the manufacturer’s instructions. For determination of total ADP and ATP as well as AMP in the cells, the same ADP Assay Kit (MilliporeSigma, MAK133) or AMP Assay Kit (Abcam, ab273275), respectively, were used. Luminescence and absorbance were measured in 96-well plates with FLUOstar Omega (BMG Labtech).

The adenosine released into the cell culture medium at 24 h lines was determined using the fluorometric Adenosine Assay Kit (Abcam, ab211094) following the manufacturer’s instructions. Fluorescence was measured in 96-well plates with FLUOstar Omega (BMG Labtech).

### Determination of mitochondrial ATP content by FRET

To image mitochondrial ATP-Mg^2+^ levels, cells were plated onto 8-well Lab-Tek chamber slides (Thermo Fisher, 154534) and transfected 48 h prior to the experiments. We used a plasmid encoding a ratiometric mitochondrial targeted ATP-Mg^2+^ mitGO-Ateam2 probe [43]. Transfections were performed using Lipofectamine 3000 following the manufacturer’s instructions. Experiments were performed in HEPES Buffered Saline Solution (137 mM NaCl, 1.25 mM MgSO_4_, 10 mM HEPES, 3 mM KCl, 2 mM NaHCO_3_, 2 mM CaCl_2_) supplemented with 5 mM glucose and 2 mM glutamate. Additions of the stimulus were made as a bolus in the same medium. Cells were excited for 100 ms at 485 ± 27 nm, and the emitted fluorescence was alternately collected through an FF495-Di03 dichroic at 520 ± 35 nm (GFP) and 567 ± 15 nm (OFP). Images were collected every 2.5 or 15 s using a filter wheel (Lambda 10-2, Sutter Instruments; Chroma) and recorded with an ORCA-Flash4.0 LT sCMOS camera (Hamamatsu) mounted on an Axiovert200 inverted microscope (Zeiss) equipped with a 63X/1.4 Plan-Apochromatic oil objective. The emission ratio GFP/OFP reflects mitochondrial levels of ATP-Mg^2+^.

ROIs were selected on single-cell fluorescence recordings and analyzed using MetaMorph (Universal Imaging) and ImageJ (NIH). For quantification, the time of the decrease in the GFP/OFP fluorescence ratio by 20% after stimulation was determined.

### Determination of phosphoribosyl pyrophosphate synthase and hypoxanthine-guanine phosphoribosyltransferase enzymatic activities

Freeze-clamped colon samples were homogenized in 8 volumes of 10 mM KH_2_PO_4_ pH 7 buffer using the Bead Mill 24 Homogenizer system (Fisherbran, 15515799). Cell pellets were homogenized in 8 volumes of 10 mM KH_2_PO_4_ pH 7 buffer using a glass-teflon potter. After 30 min incubation at 4 °C the homogenates were centrifuged at 16,000×*g* for 30 min at 4 °C. PRPP-Synthase activity was determined in the supernatant with the PRECICE® PRPP-S Assay Kit (NovoCib, K0709-04-2). HPRT activity was determined with the PRECICE® HPRT Assay Kit (NovoCib, K0709-01-2). A_340_ was measured in 96-well plates with FLUOstar Omega (BMG Labtech).

### Determination of Ca^2+^ content, Ca^2+^ retention capacity (CRC) and Ca^2+^ efflux of isolated mitochondria

Ca^2+^ content of isolated mitochondria was determined by fluorescence using the Ca^2+^ sensitive fluorescent probe Calcium-Green 5N (Thermo Fisher, C3737) (250 nM, excitation 506 nm, emission 532 nm) in a hypotonic solution (0.5 mM Tris-HCl pH 7.5) [81]. To calculate the amount of intramitochondrial Ca^2+^, a calibration curve was made with known CaCl_2_ concentrations in a hypotonic solution. Excitation/acquisition was performed three times with a 488/535 filter cube using a BMG labtech FLUOstar OPTIMA plate reader.

The calcium retention capacity (CRC) of isolated mitochondria was determined using 0.1 μM Calcium-Green 5 N (Thermo Fisher, C3737) in MSK medium (75 mM mannitol, 25 mM sucrose, 5 mM KH_2_PO_4_, 20 mM Tris-HCl, 100 mM KCl, 0.1% BSA, pH 7.4) using a BMG labtech FLUOstar OPTIMA plate reader. All experiments were carried out at 30 °C in the presence of 1 mM MgCl_2_, 5 mM succinate as respiratory substrate, and in the presence of 2 μM rotenone and 50 μM ADP. In brief, 300 µg of mitochondria were resuspended in a complete MSK medium with the Calcium-Green 5 N probe. After reaching a stable fluorescence reading, they were challenged with 10 µM CaCl_2_ additions and the Ca^2+^ uptake capacity of the mitochondria was measured as a function of the decrease in fluorescence.

To determine the Ca^2+^ efflux, the same procedure described above was carried out, but the MSK medium was supplemented with 50 µM NaCl and after two or three additions of 10 µM CaCl_2_, 0.2 µM Ruthenium Red was added.

### Cellular O_2_ consumption

Oxygen consumption rates were determined in an XF24 Extracellular Flux Analyzer (Agilent Technologies, 100867-100) in CT26 cells using palmitate as substrate. Cells were starved for 12 h in low glucose DMEM (0.05 mM glucose, 1 % FBS) and then changed to KHB media (111 mM NaCl, 4.7 mM KCl, 1.25 mM glutamine, 5 mM HEPES, pH 7.4). BSA-conjugated palmitate (1 mM sodium palmitate, 0.17 mM BSA solution) was added as the main substrate. To assess oligomycin-sensitive respiration, maximum respiration, and non-mitochondrial dependent oxygen consumption, respectively, 6 μM oligomycin (OL), 0.75 mM 2,4-dinitrophenol (DNP), and 1 μM rotenone plus 1 μM antimycin A were added.

### Isolation of colon and spleen cells

For the isolation of colon cells, colon tissue was minced and incubated with a digestion mixture, RPMI supplemented with 0.9 U ml^−^^1^ collagenase A (MilliporeSigma, 10103578001), for 2 h with shaking at 37 °C. The reaction was stopped by adding HBSS+2% FBS and centrifuged at 400×*g* for 5 min at 4 °C. The pellet was resuspended in 0.25% trypsin for 2 min, and then a mixture of 1 mg ml^−^^1^ collagenase-dispase (MilliporeSigma, 10269638001) and 1 U ml^−^^1^ DNase (MilliporeSigma, 11284932001) in HBSS was added. Finally, the reaction was stopped by adding HBSS+2% FBS and centrifuged at 400×*g* for 5 min at 4 °C. The pellet was resuspended in HBSS+2% FBS and sequentially filtered using a 70-µm filter (Falcon) and a 40-µm filter (Falcon) to remove undigested tissue. The filtrated was centrifuged at 400×*g* for 5 min at 4 °C, and the pellet was resuspended in HBSS+FBS 2% to proceed with the corresponding analyses. These cells were used for the analysis of ∆Ψm and mtROS production under basal conditions, as well as for the study of the immune system population by flow cytometry under inflammatory conditions.

Spleens from mice were minced with HBSS+2% FBS using 40-µm filters (Falcon). The homogenate was centrifuged at 400×*g* for 5 min at 4 °C and resuspended in ACK lysis buffer (0.15 M NH_4_Cl, 10 mM KHCO_3_, 0.1 mM EDTA, pH 7.2–7.4) for 5 min at room temperature, to promote lysis of erythrocytes. This reaction was stopped by adding HBSS+2% FBS and centrifuged at 400×*g* for 5 min at 4 °C, and the pellet was resuspended in HBSS+2% FBS to proceed with the corresponding analyses.

### Measurement of ΔΨm and mtROS

ΔΨm and mtROS were determined in cell lines and colonocytes by flow cytometry staining with 500 nM TMRM (Invitrogen, T668) and 5 μM MitoSOX (Invitrogen, M36008) probes, respectively [29]. DAPI (diamidino-2-fenilindol) (Merck KGaA, 268298) was used to exclude dead cells. The fluorescence intensity of at least 10,000 events was determined in a FACS Canto II cytometer (Becton Dickinson) at Cytometry Facilities (CBMSO, Spain) and analyzed using the Flow Jo V10 software (Tree Star). The specificity of TMRM staining was assessed by the addition of 5 μM DNP to collapse ΔΨm or 5 μM oligomycin. The specificity of MitoSOX staining was assessed by the addition of 5 μM oligomycin or 5 μM antimycin A.

### Determination of mtDNA copy number and mtDNA oxidative damage

Total genomic DNA (nuclear and mitochondrial) was extracted from the colon with phenol:chloforofom:isoamyl alcohol (25:24:1) method. Mitochondrial/nuclear DNA (mt/nDNA) ratio was quantified with Fast SYBR Master Mix in an ABI PRISM 7900HT sequence detection system (Thermo Fisher) at the Genomics and NGS Core Facility (CBMSO, Spain). Thermal cycling conditions were as follows: initial denaturation of 20 s at 95 °C, 40 amplification cycles of 1 s at 95 °C, and 20 s at 60 °C each, followed by a dissociation curve analysis to detect possible nonspecific amplification. Standard curves with serial dilutions of pooled DNA were used to assess the amplification efficiency of the primers and to establish the dynamic range of DNA concentration for amplification, which was 8 ng per run. The relative copy number of mtDNA molecules was determined with the comparative ΔΔC_T_ method using *nActb*, *nAtp5b* and *nB2M* as nuclear genes and *mt-12S, mt-16S* and *mtND4* as mitochondrial genes. Primers used are listed in Supplementary Table S1.

For the determination of DNA oxidative damage, total and mtDNA were extracted from colon extracts or colon mitochondria as previously described. The 8-OH-dG content was quantified using EpiQuik™ 8-OHdG DNA Damage Quantification Direct Kit (Fluorometric) (EpigenTek), following the manufacturer’s instructions.

### RNA extraction and RT-PCR analysis

RNA was extracted and purified from the colon with Trizol reagent (Invitrogen, 15596026) according to the manufacturer’s instructions. Purified RNA was quantified with a Nanodrop Spectrophotometer (Thermo Fisher). Reverse transcription reactions were performed using 1 μg of total RNA and the High Capacity cDNA Reverse Transcription Kit (Thermo Fisher, 4368814). Real-time PCR was done with Fast SYBR Master Mix (Thermo Fisher, 4385616) in an ABI PRISM 7900HT sequence detection system (Thermo Fisher) from the Genomics and NGS Core Facility (CBMSO). Thermal cycling conditions were as follows: initial denaturation of 20 s at 95°C, 40 amplification cycles of 1 s at 95°C, and 20 s at 60°C each, followed by a dissociation curve analysis. Standard curves with serial dilutions of pooled cDNA were used to assess the amplification efficiency of the primers and to establish the dynamic range of cDNA concentration for amplification, which was 3 ng of input cDNA per run. Primers used to amplify the target genes are listed in Supplementary Table S1. The relative expression of the mRNAs was determined with the comparative ΔΔC_T_ method with β-actin and GAPDH as housekeepings.

### Histochemistry and immunofluorescence microscopy

Tissue sections were fixed in 4% PFA and included in paraffin or OCT blocks to be cut into 5 or 10 μm slices, respectively. Paraffin sections were stained with hematoxylin/eosin. To assess infiltration of Gram+ bacteria, colon slices were stained with a GRAM STAIN kit (MilliporeSigma, HT90T-1KT). For immunofluorescence, OCT sections were incubated with the primary antibodies (listed in Supplementary Table S2). Nuclei were counter-stained with DAPI (diamidino-2-fenilindol) (Merck KGaA, 268298) reagent. Images were processed in an Axioskop2 plus vertical microscope (Zeiss) coupled to a DMC6200 camera (Leica) at the Optical and Confocal Microscopy Facility (CBMSO, Spain). Cellular fluorescence was analyzed by confocal microscopy in a high acquisition speed and sensibility A1R+ microscope (Nikon). All images were processed with ImageJ software (National Institutes of Health).

### Multiplexed quantitative analysis of cytokines

Mice were sacrificed with CO_2_ euthanasia and ~300 μl of blood were aspirated from the heart of the animals. After 30 min of blood coagulation at room temperature, the samples were centrifuged for 1.5 min at 10,600×*g* in Microtainer® SSTTM (BD) tubes and the serum was collected and stored at −80 °C. Colon tissue was collected after sacrifice and homogenized in the special lysis buffer medium (PBS pH 7.4, 2 mM MgCl_2_, protease inhibitor cocktail without EDTA, Benzonase® Nuclease) with the DWK Life Sciences Kimble™ Kontes™ Pellet Pestle™ system. Extracts were incubated for 5 min on ice and then subjected to 10 min centrifugation at 10,000×*g* at 4 °C. The levels of cytokines in the serum and colon from mice were analyzed following the protocol of Mouse T Cell Kit, MILLIPLEX MAP Assay (Merck, MHSTCMAG-70K).

### Mouse treatments

FITC-dextran Permeability Assay. To assess the permeability of the intestinal epithelial barrier, we used the FITC-dextran (MilliporeSigma, FD4-250MG). Mice were first starved for 8 h and then given 44 mg kg^−1^ of body weight of FD4 dissolved in water by oral gavage. Serum was collected 4 h later and the amount of FD4 was determined by spectrofluorometry with the 485 nm excitation wavelength filter and 528 nm emission wavelength filter using a FLUOstar Omega luminometer (BMG Labtech).

#### DSS-induced colon inflammation

Four-month-old mice were treated with 2% DSS (PanReac AppliChem, A3261,0250) added to the drinking water to induce acute intestinal inflammation [17]. The weight of mice was recorded every day, and the colon length was determined after euthanasia.

#### ADORA2B agonist/antagonist treatment in LPS-induced inflammation

Four-month-old mice were treated by intraperitoneal injection for 7 days with ADORA2B agonist BAY60-6583 (MilliporeSigma, SML1958-5MG) (1 mg kg^−1^) or antagonist PSB-603 (MilliporeSigma, SML1983) (5 mg kg^−1^). On the fifth day, lipopolysaccharide (LPS) from *Escherichia coli* (MilliporeSigma, L2630) (8 mg kg^−1^) was intraperitoneally injected. Weight determination was daily determined until the day of sacrifice as a measure of animal welfare.

### Determination of immune system subsets by flow cytometry

Cells isolated from the colon and spleen were stained in p96 V-bottom plates. Each well contained 2–5 × 10^5^ cells, and all procedures were carried out at 4 °C using PBS staining buffer supplemented with 1% BSA and 0.02% sodium azide. For all flow cytometry staining, cells were incubated with anti-CD16/32 antibodies in a staining buffer for 20 min. The plate was then centrifuged at 400×*g* for 2 min at 4 °C, the supernatant discarded, and the cells washed with PBS supplemented with 1% BSA. Antibody cocktails were then added to the samples, and the cells were incubated at 4 °C for an additional 30 min. Antibodies used are listed in Supplementary Table S2. Cells were washed with PBS+1% BSA and resuspended in a final volume of 200 µl to analyze the samples by flow cytometry.

For intracellular lymphocyte staining, cells were incubated in vitro for 6 h with plate-coated with anti-CD3, phorbol 12-myristate 13-acetate (50 ng ml^−1^; MilliporeSigma), ionomycin (500 ng ml^−1^; MilliporeSigma) and Brefeldin A (2 μg ml^−1^; MilliporeSigma). Following extracellular staining, cells were washed and resuspended in permeabilization-fixation solution (BD Cytofix/Cytoperm Kit; BD Pharmingen, RRID: AB_2869008), and intracellular cytokine staining was performed with appropriate fluorescently labeled antibodies (listed in Supplementary Table S2) following the manufacturer’s protocol. Flow cytometry experiments were performed using a FACS Canto II flow cytometer (Becton Dickinson) at the Cytometry Facility (CBMSO, Spain). All flow cytometry data analyses were performed using Flow Jo V10 software (Tree Star Inc).

For analysis of the different lymphocytic subsets, the following cocktail of antibodies was used: for CD4^+^ cells anti-CD45, anti-CD3 and anti-CD4; for CD8^+^ cells anti-CD45, anti-CD3 and anti-CD8; for Th1 subset anti-CD45, anti-CD4 and anti-IFNγ; for Th17 subset anti-CD45, anti-CD4 and anti-IL-17; for Treg subset anti-CD45, anti-CD4 and anti-FOXP3; for M1 macrophages anti-CD45, anti-CD11b, anti-Ly-6C and anti-F4/80; for neutrophils anti-CD45, anti-CD11b and anti-Ly-6G.

### Proteomic analysis

Quantitative proteomic analysis of colon tissue extracts from CRL and IF1-KO mice was performed with isobaric tags for relative and absolute quantitation (iTRAQ, AB Sciex, 4383502) labeling method coupled to tandem mass spectrometry (MS/MS) as previously described [18]. Quantitative proteomic analysis of isolated colon mitochondria from CRL and IF1-KO mice (*n* = 3) was performed with Tandem Mass Tag™ 6-plex (TMTsixplex, Thermo Fisher, 90061) labeling method coupled to tandem mass spectrometry (MS/MS). In-gel digestion of protein extracts, iTRAQ/TMTsixplex labeling, reverse phase-liquid chromatography-MS/MS analysis and protein identification and quantitation were performed by the Proteomic Facility (CBMSO, Spain).

#### In-gel digestion

Protein extracts (30 μg) were suspended in a volume of up to 50 µl of sample buffer and then applied onto 1.2-cm wide wells of a conventional SDS-PAGE gel (0.75 mm thick, 4% stacking, and 10% resolving). The run was stopped as soon as the front entered 3 mm into the resolving gel to concentrate the proteome. The unseparated protein bands were visualized by Coomassie staining, excised, cut into cubes (2 × 2 mm), and placed in 0.5 ml microcentrifuge tubes. The gel pieces were destained in acetonitrile:water (ACN:H_2_O, 1:1), were reduced and alkylated (disulfide bonds from cysteinyl residues were reduced with 10 mM DTT for 1 h at 56 °C, and then thiol groups were alkylated with 10 mM iodoacetamide for 30 min at room temperature in darkness) and digested in situ with sequencing grade trypsin (Promega) [18]. Gel pieces were shrunk by removing all liquid using sufficient ACN. Acetonitrile was pipetted out and the gel pieces were dried in a speedvac. The dried gel pieces were re-swollen in 100 mM Tris-HCl pH 8, 10 mM CaCl_2_ with 60 ng/µl trypsin at 5:1 protein:enzyme (w/w) ratio. The tubes were kept in ice for 2 h and incubated at 37 °C for 12 h. Digestion was stopped by the addition of 1% TFA. Whole supernatants were dried down and then desalted onto OMIX Pipette tips C18 (Agilent Technologies) or ZipTip C18 Pipette tips (MilliporeSigma) until the mass spectrometric analysis.

#### Reverse phase-liquid chromatography RP-LC–MS/MS analysis

The desalted protein digest was dried, resuspended in 10 µl of 0.1% formic acid and analyzed by RP-LC-MS/MS in an Easy-nLC II system coupled to an ion trap LTQ-Orbitrap-Velos-Pro hybrid mass spectrometer (Thermo Fisher). The peptides were concentrated (on-line) by reverse phase chromatography using a 0.1 × 20 mm C18 RP precolumn (Thermo Fisher) and then separated using a 0.075 × 250 mm C18 RP column (Phenomenex) operating at 0.3 μl min^−1^ (proteomics). For proteomics, peptides were eluted using a 90-min dual gradient, and the gradient profile was set as follows: 5–25% solvent B for 68 min, 25–40% solvent B for 22 min, 40–100% solvent B for 2 min and 100% solvent B for 18 min (Solvent A: 0.1% formic acid in water, solvent B: 0.1% formic acid, 80% acetonitrile in water). ESI ionization was done using a Nano-bore emitters Stainless Steel ID 30 μm (Proxeon) interface at 2.1 kV spray voltage with S-Lens of 60%. The Orbitrap resolution was set at 30.000 [18]. Peptides were detected in survey scans from 400 to 1600 amu (1 μscan), followed by 20 data-dependent MS/MS scans (Top 20), using an isolation width of 2 u (in mass-to-charge ratio units), normalized collision energy of 35–40%, and dynamic exclusion applied during 60 s periods. Charge-state screening was enabled to reject unassigned and singly charged protonated ions.

#### Quantitative data analysis

Peptide identification from raw data was carried out using the PEAKS Studio X+ search engine (Bioinformatics Solutions Inc.). Database search was performed against uniprot-mus-musculus.fasta (55398 entries; UniProt release 01/2020) (decoy-fusion database). The following constraints were used for the searches: tryptic cleavage after Arg and Lys (semispecific), up to two missed cleavage sites, and tolerances of 20 ppm for precursor ions and 0.05 Da for MS/MS fragment ions. The searches were performed, allowing optional Met oxidation and Cys carbamidomethylation and fixed iTRAQ/TMT6plex reagent labeling at the N-terminus and lysine residues. False discovery rates (FDR) for peptide spectrum matches (PSM) were limited to 0.01. Only those proteins with at least two distinct peptides and at least one unique peptide being discovered from LC/MS/MS analyses were considered reliably identified and sent to be quantified.

Quantitation of iTRAQ/TMT labeled peptides was performed with Proteome Discoverer 1.4 and PEAKS 8 software or PEAKS Studio X+ search engine, selected “Reporter Ion Quantification iTRAQ/TMT” under the “Quantifications” options. We use Auto normalization mode that calculates a global ratio from the total intensity of all labels in all quantifiable peptides. The -10LgP (corresponding to 1% FDR), Quality (17.5) and Reporter Ion Intensity (3.8e4) were used for Spectrum filter and Significance (20 and PEAKSQ method) was used for peptide and protein abundance calculation. For protein quantification, we consider protein groups for peptide uniqueness.

Ingenuity Pathway Analysis (IPA, Qiagen) was used to infer the canonical pathways and biological functions significantly enriched in the protein list and predict their activation state. Fisher’s exact test identified significantly enriched pathways and a *z*-score was calculated to predict whether the pathway was activated or inhibited.

### Untargeted metabolomic analysis

Untargeted metabolomic analyses of the serum and colon samples from control and IF1-KO mice were performed by UPLC-MS.

#### Metabolite extraction

For this, 20 µl of serum was mixed with 100 µl of ice-cold methanol with 2 µg ml^−1^ N-Benzoyl-L-tyrosine ethyl ester as internal standard (IS). The resulting mixtures were vortexed for 15 min at 4 °C, incubated at −20 °C for 5 h and then centrifuged at 4 °C and 14,000×*g* for 10 min. Supernatants were evaporated to dry in a SpeedVac. The dry residues were reconstituted in acetonitrile/water (50:50, v/v) and analyzed by UPLC-MS. Colon tissues were grinded with liquid nitrogen. For each 20 µg of colon tissue, 300 μl ice-cold methanol-water (2:1, v/v) and 200 μl chloroform with IS were sequentially added. Three cycles of freezing/thawing and mechanical homogenization with a Bead Mill 24 Homogenizer system were performed. The mixture was centrifuged at 14,000×*g* and 4 °C for 10 min, and the upper phase was analyzed by UPLC-MS. A pooled “quality control” (QC) sample was prepared by mixing equal volumes from all extracted samples from either serum or colon. Blank samples were prepared by applying the same sample preparation protocol to identify those background ions that were associated either with the extraction solvents and/or with UPLC-MS analysis.

#### Data acquisition

Analyses were performed in a Waters Acquity UPLC system hyphenated to a Bruker maXis II UHR-QTOF mass spectrometer. Chromatographic separation was performed using an ACQUITY UPLC BEH C18 (2.1 mm × 50 mm 1.7 μm) column (Waters). Mobile phase A was 0.1% formic acid in water, and mobile phase B was 0.1% formic acid in acetonitrile. For negative mode, the mobile phase modifier was 0.01% formic acid. Column and autosampler temperatures were set at 40 and 6 °C, respectively. The sample injection volume was 2 μl. UHR-QTOF mass spectrometer instrument was operated in both positive and negative ESI modes in separate analyses. Mass detection was run in the MS scan mode from *m/z* 20 to 2000. Samples were analyzed in randomized order. QCs were injected at the beginning of the analysis and intercalated to assess optimal instrument performance across the sample set in terms of retention time, peak area and mass accuracy.

#### Data processing, statistical analysis and metabolite identification

Raw LC-MS data were converted to mzXML format using ProteoWizard software [82] and processed with the XCMS and CAMERA software package (Scripps Institute for Metabolomics). This software provides retention time alignment, metabolite feature detection, feature matching, peak integration, adduct and isotope annotation. Peak areas integrated by XCMS were normalized with the IS added to each sample. Differences between groups were studied using univariate and multivariate statistical analysis using MetaboAnalyst 3.0 [83]. For multivariate analyses, the data were log-transformed and Pareto-scaled to remove the offsets and adjust the importance of low and high abundance features to an equal level. Non-supervised Principal component analysis (PCA) was performed in search of discriminant metabolomic patterns and for outlier detection. Supervised multivariate data analysis was performed by a partial least-squares discriminate analysis (PLS-DA) to further differentiate the contributions of particular metabolites to the separations of the different sample groups. The corresponding *variable importance in the projection* (VIP) values were calculated in the PLS-DA model. The potentially significant biochemical variables (metabolite signals) were selected when they met the requirements of VIP of 1 and above in the PLS-DA model. Univariate analysis was performed by applying a *t*-test or the Mann–Whitney U test depending on the normality of the distribution of each metabolite. Fold change was calculated for each metabolite in order to estimate the variation in the abundance of the metabolites within each comparison. Metabolite variables with VIP ≥1 and with statistically significant differences (*p* < 0.05) were submitted to tentative identification. Metabolite tentative identification was performed by the query of the exact mass of the detected features against online databases (HMDB and Metlin) within a ±10 ppm mass range. Additionally, the identified metabolites were imported into MetaboAnalyst 3.0 to determine which pathways are involved in the comparative analysis of IF1-KO vs control samples.

### Targeted metabolomic analysis

Freeze-clamped colon tissues were precipitated in six volumes of 6% perchloric acid, incubated on ice for 1 h and then centrifuged at 11,000×*g* for 5 min at 4 °C to obtain a protein-free supernatant. The supernatants were neutralized with 20% KOH and centrifuged at 11,000×*g* and 4 °C for 5 min to sediment the KClO_4_ salt. Samples were vortexed, incubated in ice for 30 min, and centrifuged at 16,100×*g* for 5 min at 4 °C. Sample analysis was performed using a UHPLC system (1290 Infinity II system, Agilent Technologies, Waldbronn), coupled to a 6546 LC/QTOF (Agilent Technologies) with an ESI ion source operated in negative and positive ionization modes. An XBridge BEH Amide XP Column (130 Å, 2.5 µm, 2.1 mm×100 mm, SKU: 176002589, Waters) was used for hydrophilic liquid chromatography (HILIC) interaction. Briefly, the mobile phases for the analyses in negative ionization mode: mobile phase A was ammonium acetate 10 mM (pH 9.0) with InfinityLab deactivator additive 2.5 mM (Agilent, P-N. 5191-4506) and mobile phase B was 10 mM ammonium acetate (pH 9.0) in H_2_O/ACN (15:85, v/v, using weighed acetonitrile) with 2.5 mM InfinityLab deactivator additive. The mobile phases for the analyses in positive ionization mode: mobile phase A 10 mM ammonium formate in water with 0.1% formic acid and 2.5 mM InfinityLab deactivator additive and mobile phase B was 10 mM ammonium formate in H_2_O/ACN (1:9, v/v, using weighed acetonitrile) with 0.1% formic acid and 2.5 mM InfinityLab deactivator additive. For negative mode, the flow was constant at 0.250 ml min^−1^ and the columns were kept at 50 °C. The chromatographic gradient started at 96% B and was kept for 2 min. B was decreased to 88% at minute 5.5 and kept until minute 8.5. At minute 9, the percentage of B was 86 and was kept until minute 14. B was decreased again to 82% at minute 17 and then to 65% at minute 23 and maintained until minute 24. At 24.5 min, the percentage of B was restored to initial conditions for the equilibration of the column until minute 29. For positive mode, the flow was constant at 0.250 ml min^−1^ and the temperature of the column was 25 °C. The chromatographic gradient started at 98% B and was kept for 3 min. B was decreased to 70% at minute 11, then to 60 at minute 12 and finally to 5 at minute 16 and was kept for 2 min. At minute 19, the percentage of B was restored to initial conditions for the equilibration of the column until minute 20. The injection volume was 2 µl, stacked between two 8 µl bands of ACN for those samples analyzed in negative ionization mode. For positive ionization mode, the injection volume was 2 µl. Data were collected in positive and negative ionization mode in separate analyses, operated in full scan mode for both analyses. The capillary voltage was set to 3500 V for negative mode and 3000 V for positive. The drying gas flowed at 13 l min^−1^ at 225 °C for negative mode, and 6 l min^−1^ at 225 °C for positive mode. The gas nebulizer was set to 35 psi for negative and 40 psi for positive. Fragmentor voltages were 125 V in both modes. Two reference masses were used per ionization mode in order to provide a constant mass correction: reference masses used for positive mode were 1033.9881 and 112.9855 and for negative mode were 922.009798 and 121.050873. Raw data obtained was first inspected in MassHunter Qualitative Analysis 10 (Agilent Technologies). The peak areas were integrated in MassHunter TOF Quantitative Analysis. This data was finally treated in Excel for the calculations of averages and RSD values.

### Quantification and statistical analysis

The results shown are the mean ± SEM. All tests were performed in a non-blinded fashion. Statistical analyses were performed by Student’s *t*-test or one-way ANOVA with Tukey’s post hoc test. Survival curves were derived from Kaplan–Meier estimates and compared by log-rank test. Statistical analyses were performed using Excel Microsoft 365 and GraphPad Prism 7. Values of *p* < 0.05 were considered statistically significant. Statistical details and methods used in each experiment can be found in the figure legends. *p-*values are provided in figure legends (*p < 0.05, **p < 0.01, ***p < 0.001). The *n* used in each statistical test is indicated in the figure legends, and when not specified, *n* refers to the animals or sample size per genotype.

## Supplementary information

Original Data File

Supplementary Information

Reproducibility check list

filled CDD author contribution form

## Data Availability

Proteomics data have been deposited in the PRIDE database with accession numbers of PXD038107, PXD038108, PXD038109 and PXD038151. All other data are available from the corresponding author upon reasonable request.
