# Supplementary material for: IF1 ablation prevents ATP synthase oligomerization, enhances mitochondrial ATP turnover and promotes an adenosine-mediated pro-inflammatory phenotype
Source: Cell Death Dis. 2023 Jul 12;14(7):413. doi: 10.1038/s41419-023-05957-z (PMC10336053; doi:10.1038/s41419-023-05957-z)

Original data files:

Supplemental Material to Fig 1b, i (original blots)

b

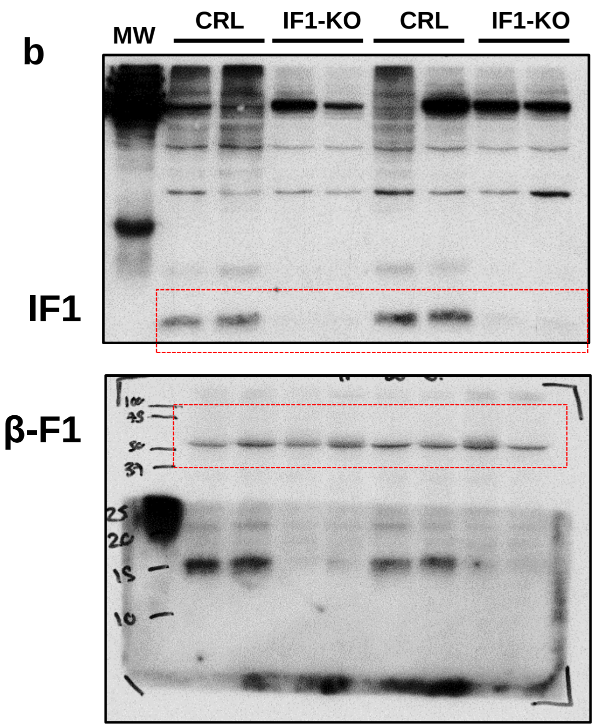

i

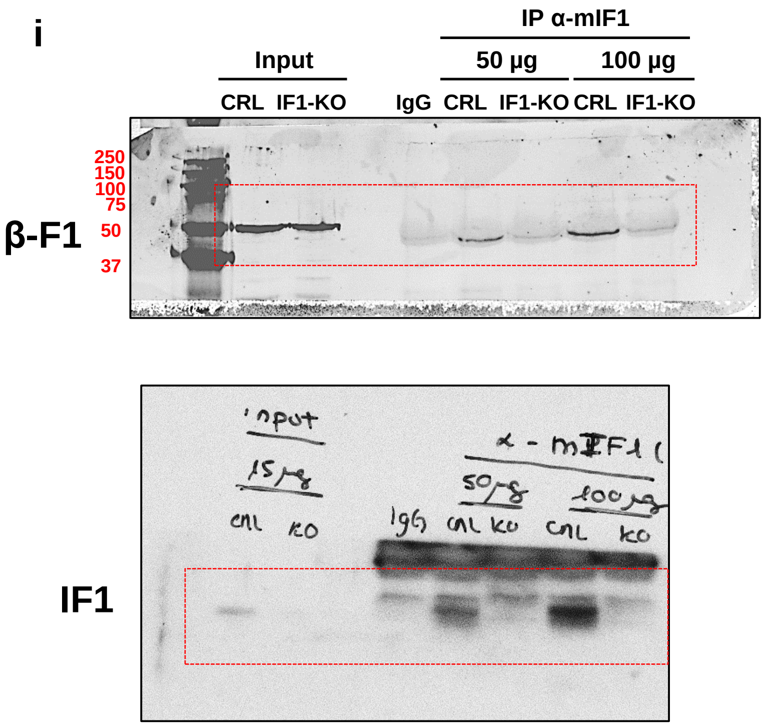

g

Supplemental Material to Fig 2g (original blots)

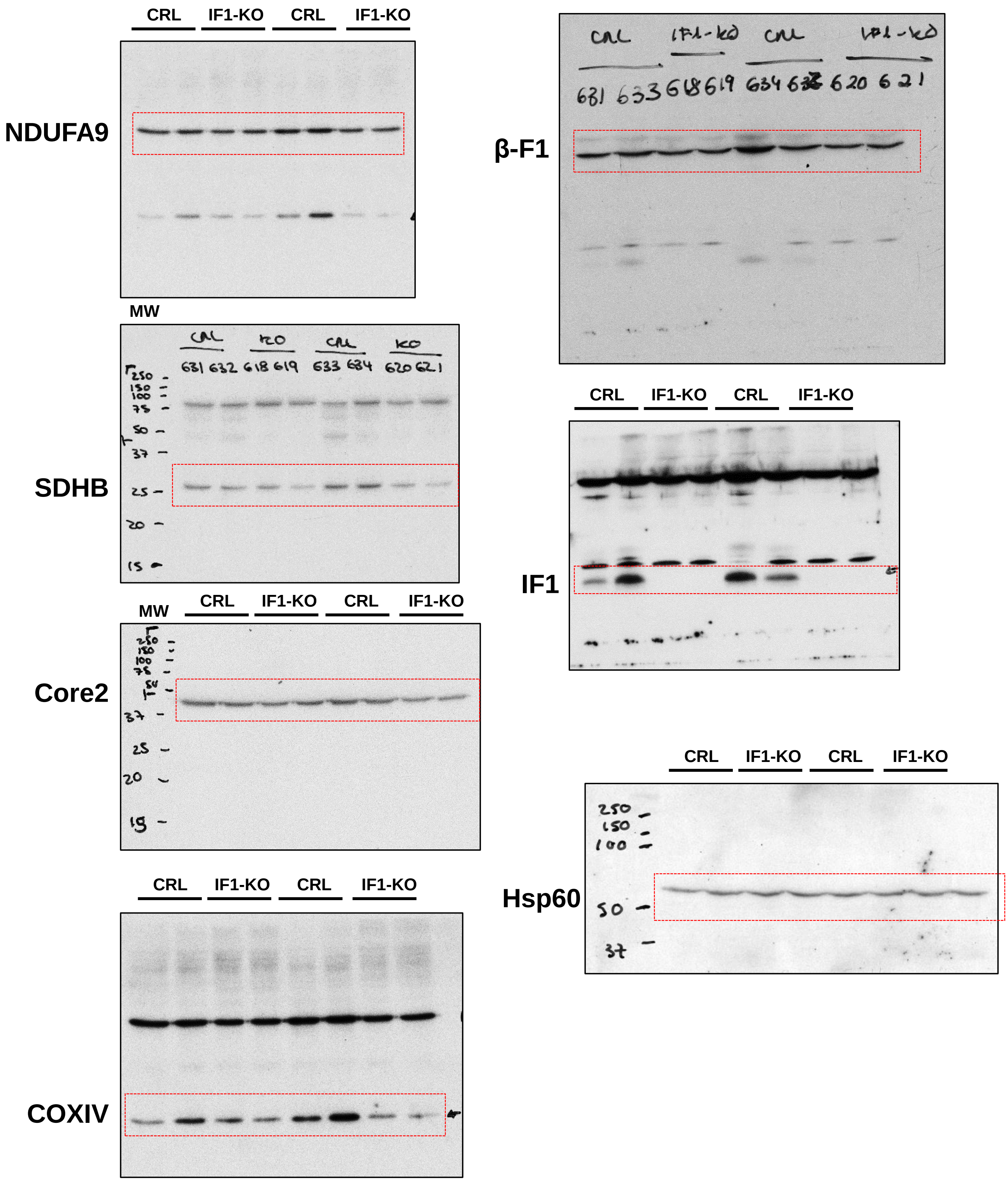

Supplemental Material to Fig 3c (original blots)

c

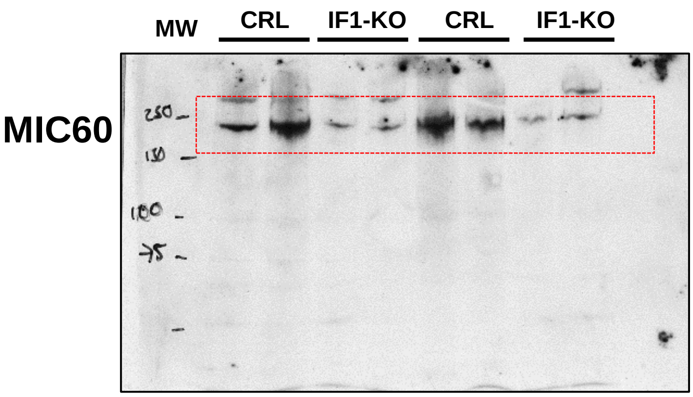

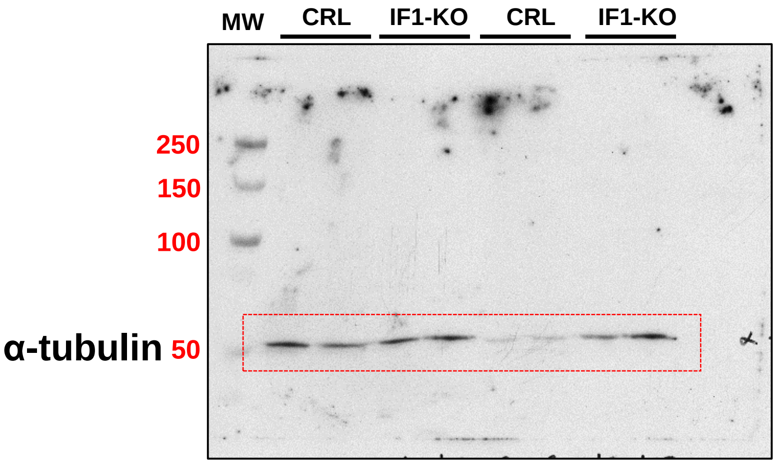

Supplemental Material to Fig 4k (original blots)

k

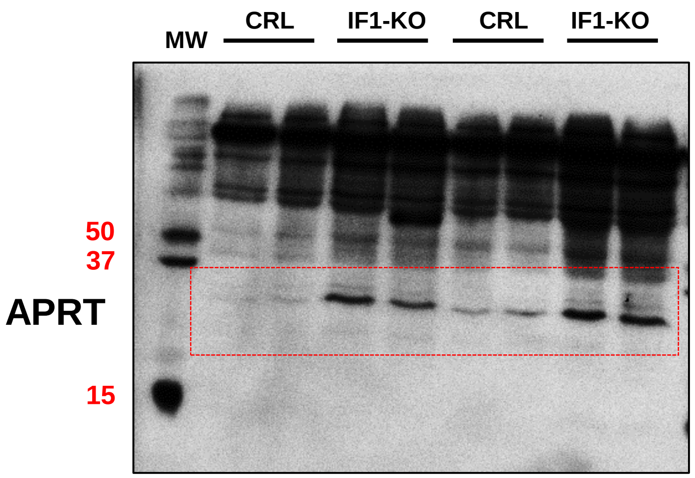

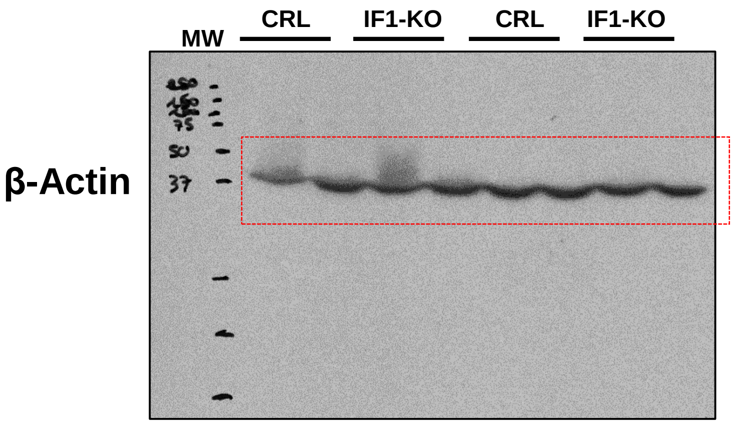

Supplemental Material to Fig 5a, i (original blots)

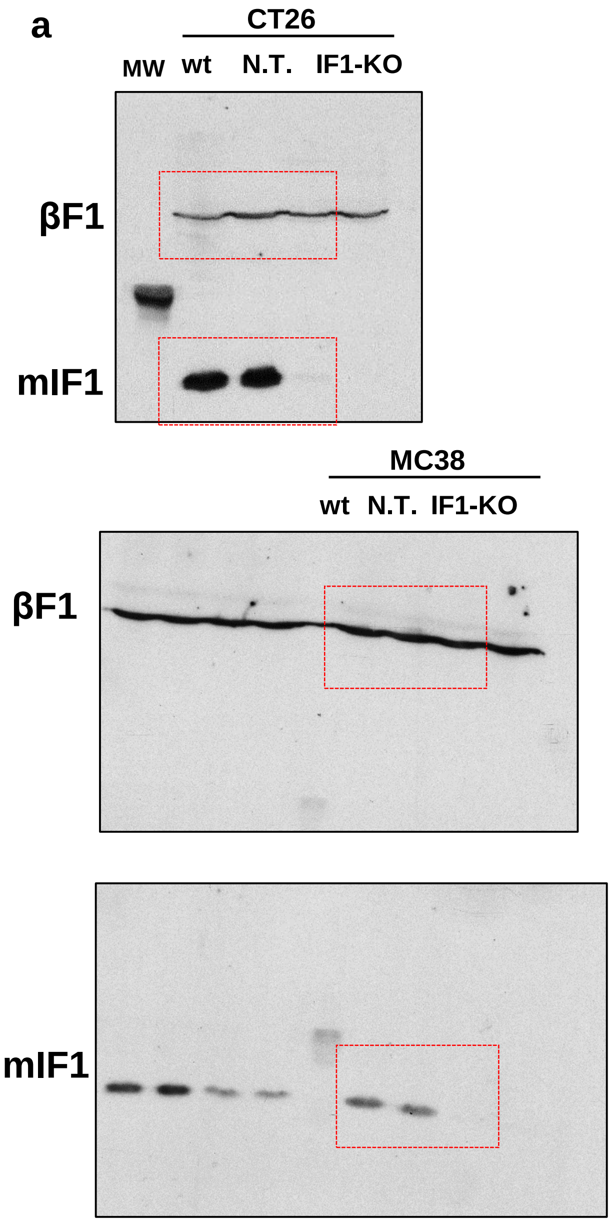

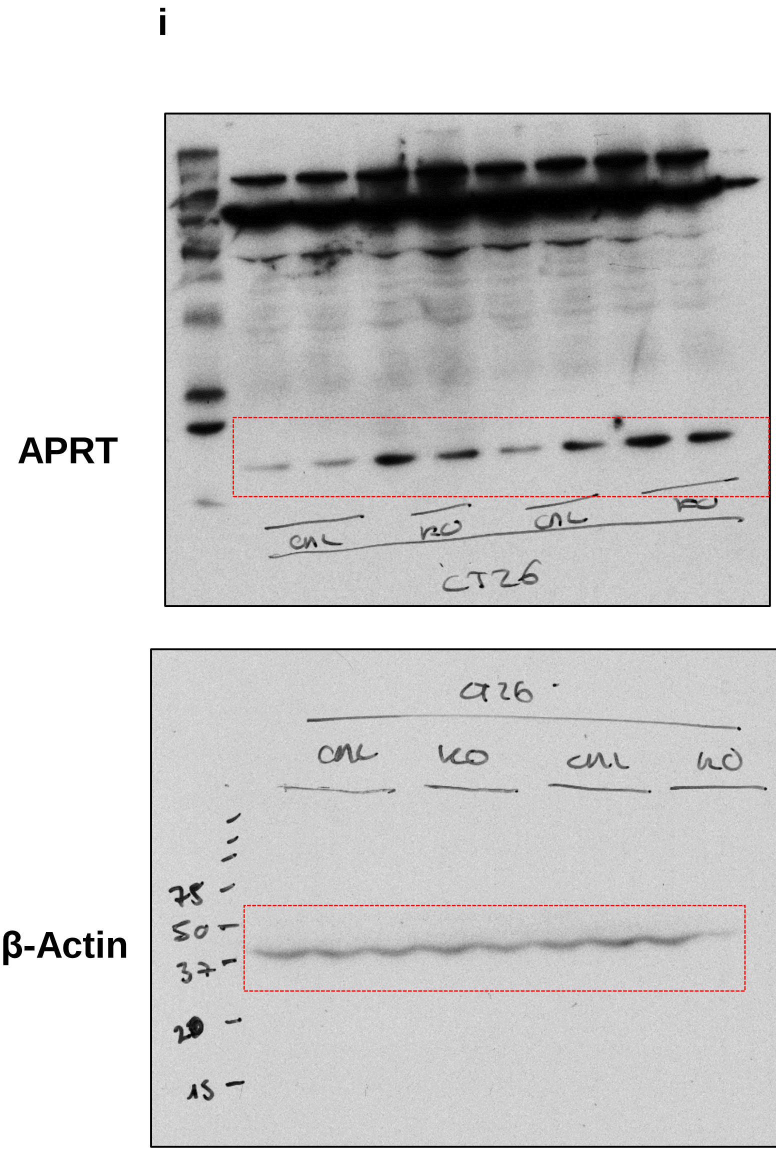

Supplemental Material to Supplemental Fig S1b-c (original blots)

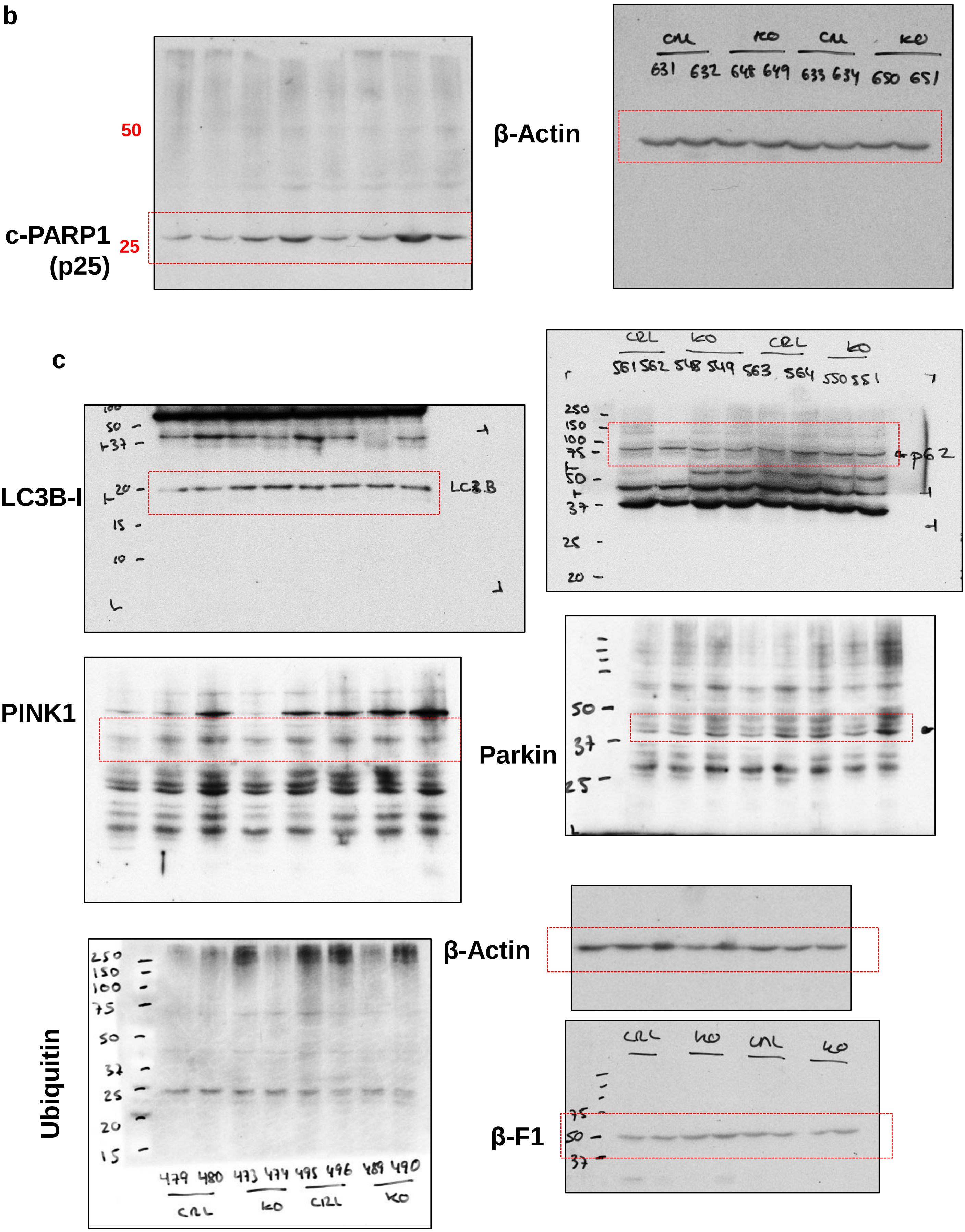

Supplemental Material to Supplemental Fig S1f (original blots)

f

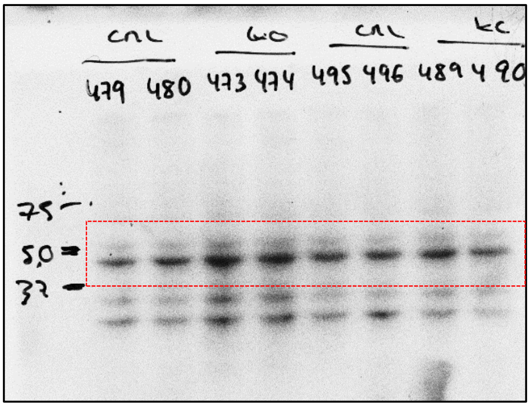

YMEL1

50
37

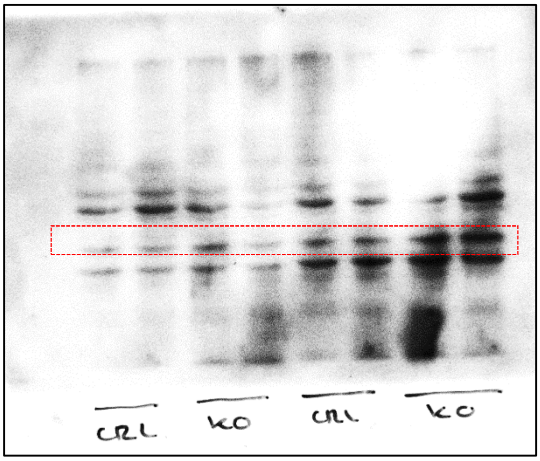

OMI

CRL IF1-KO CRL IF1-KO

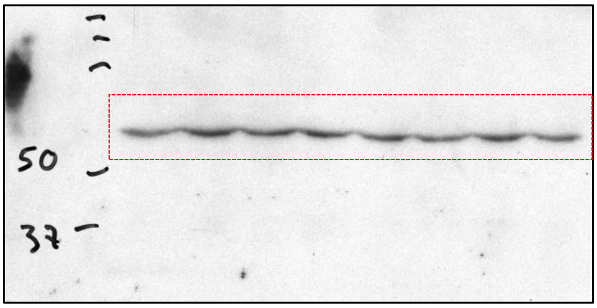

Hsp60

Supplemental Material to Supplemental Fig S2b-c (original blots)

b

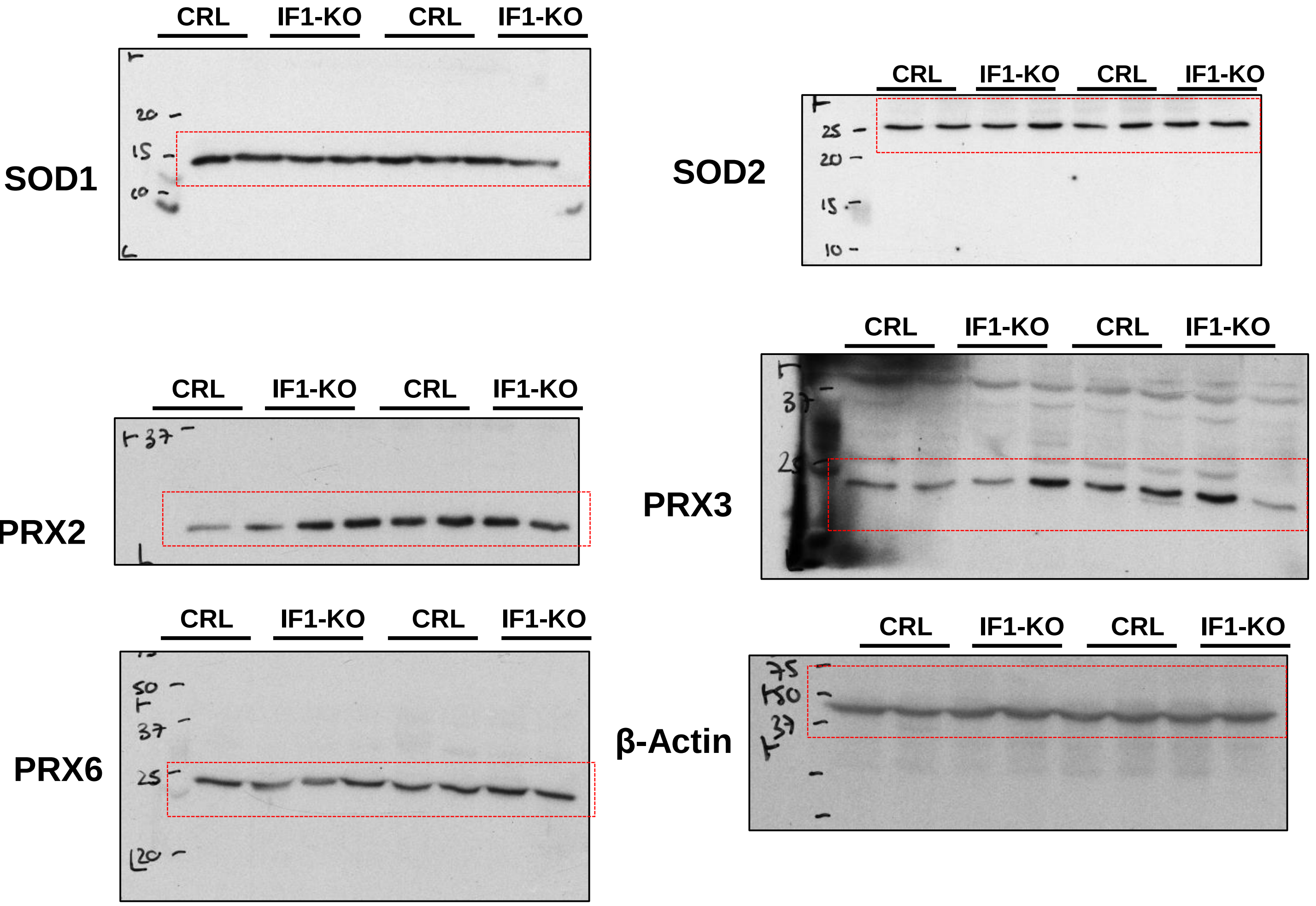

c

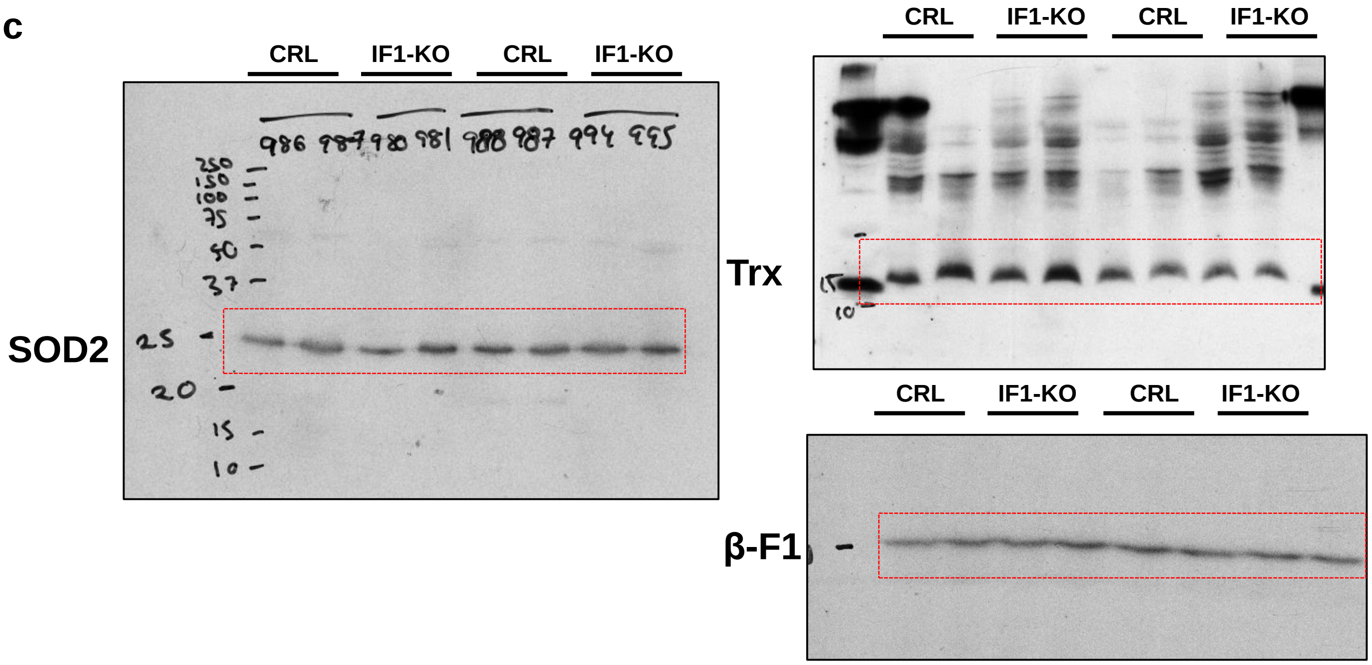

Supplemental Material to Supplemental Fig S2e-f (original blots)

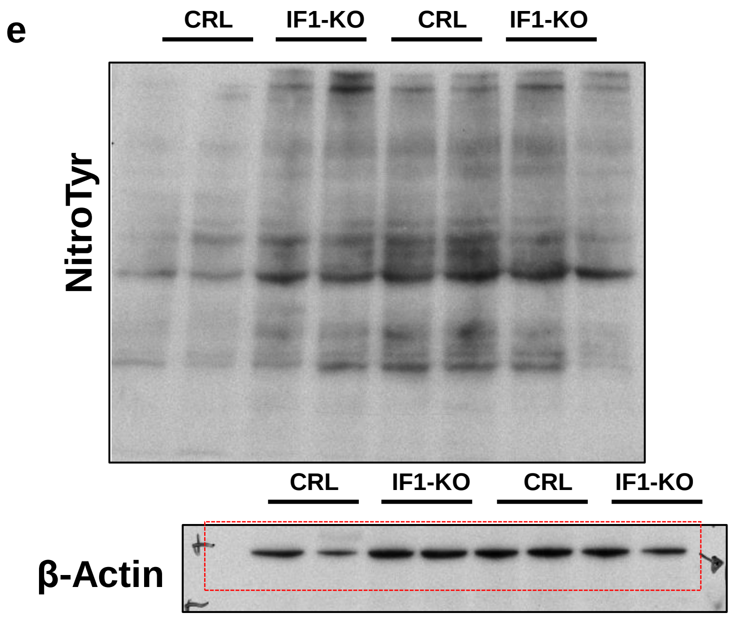

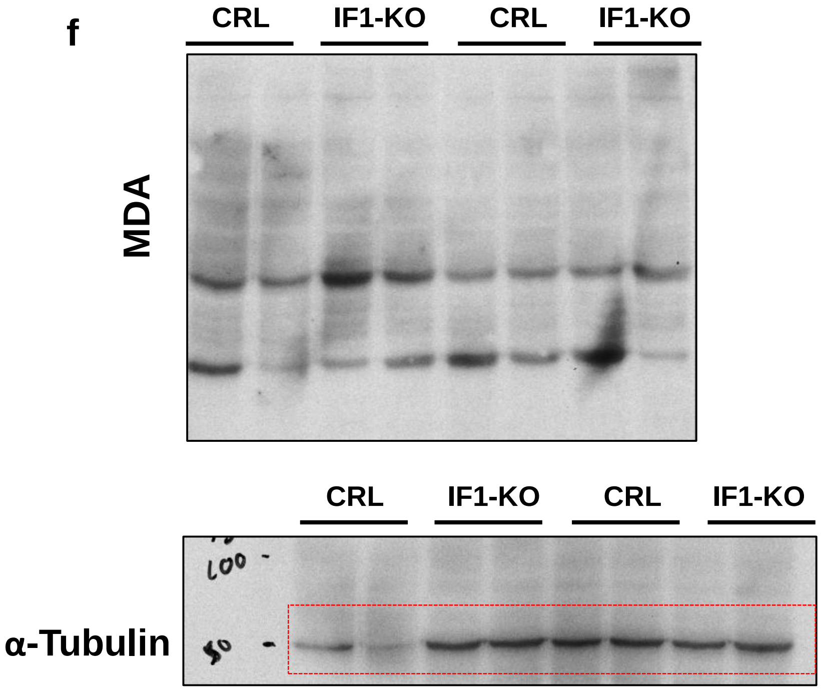

Supplemental Material to Supplemental Fig S3f-g (original blots)

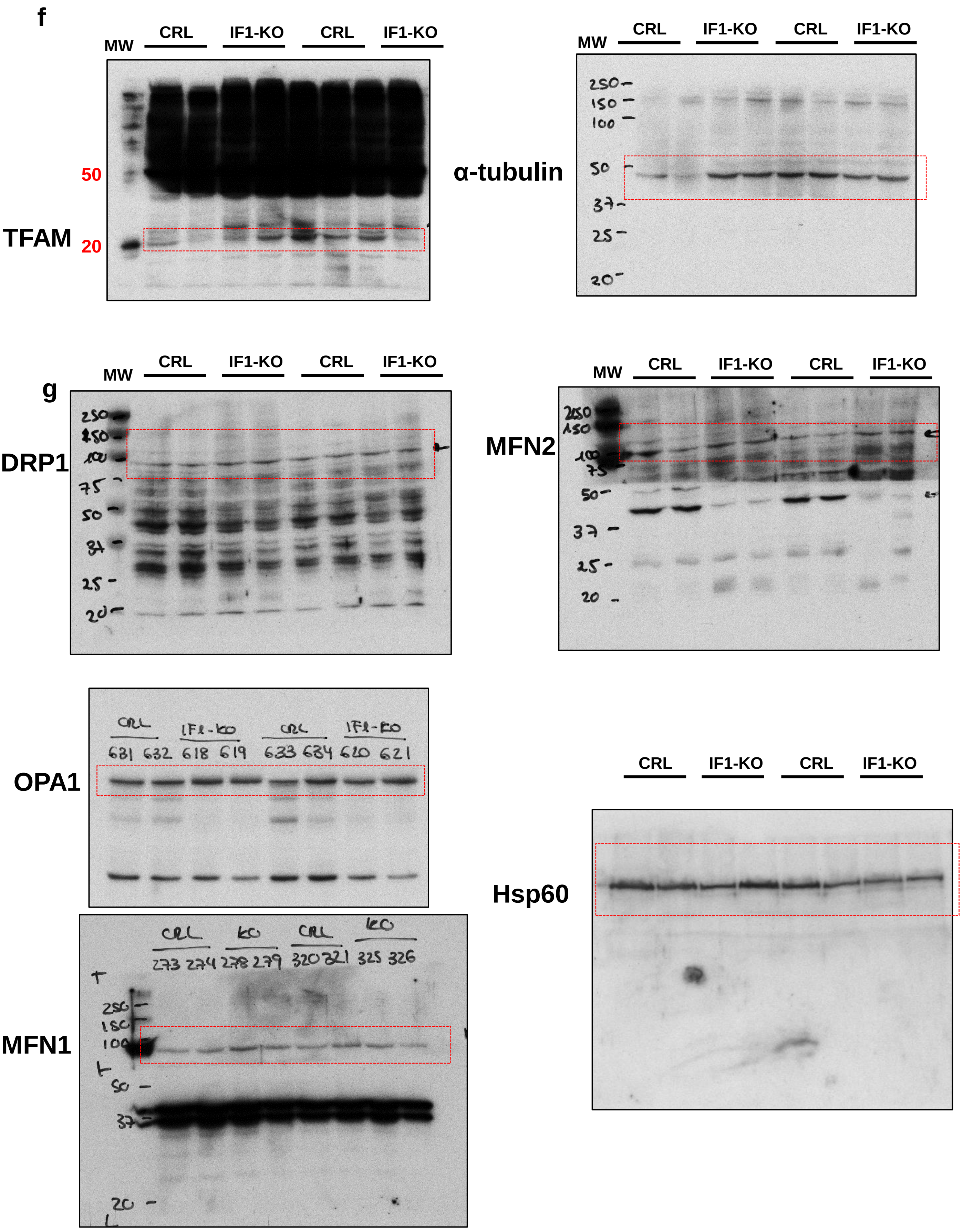

Supplemental Material to Supplemental Fig S3h (original blots)

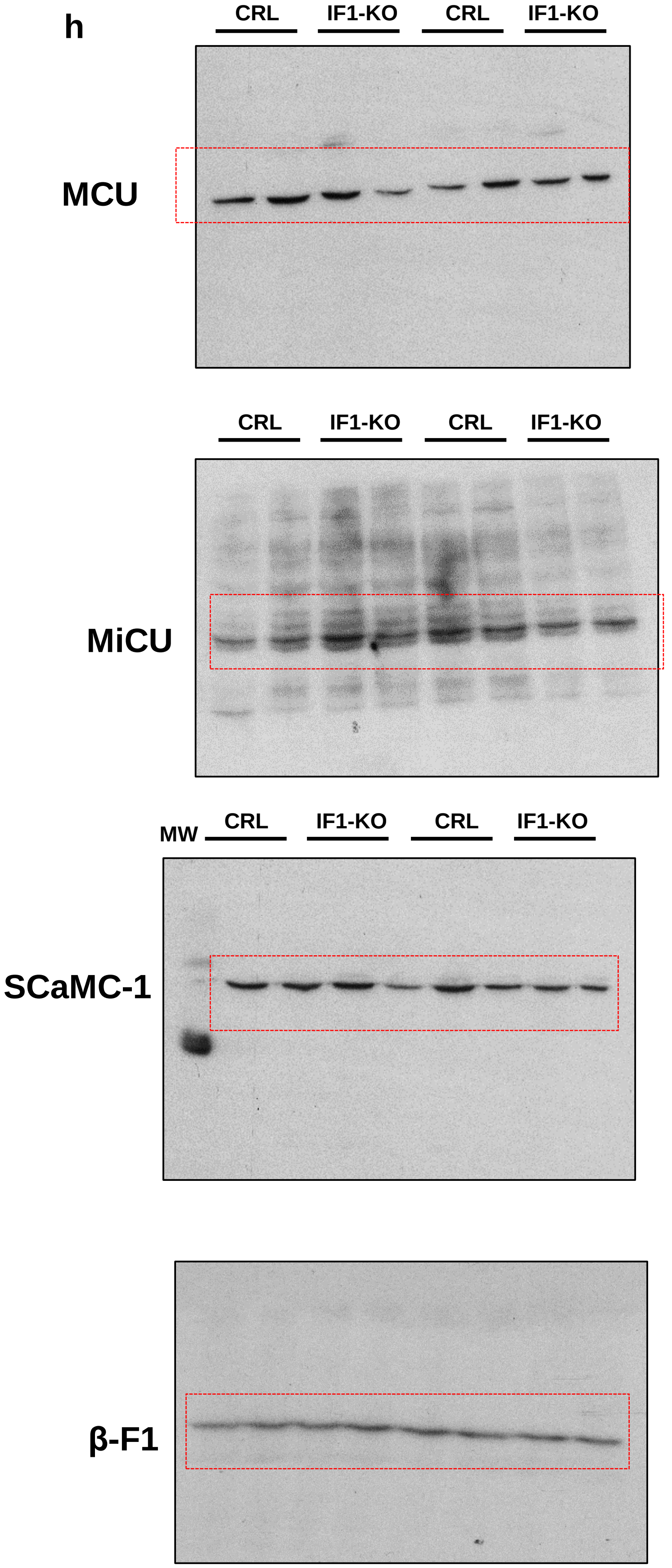

Supplemental Material to Supplemental Fig S5f (original blots)

f

MW

APRT

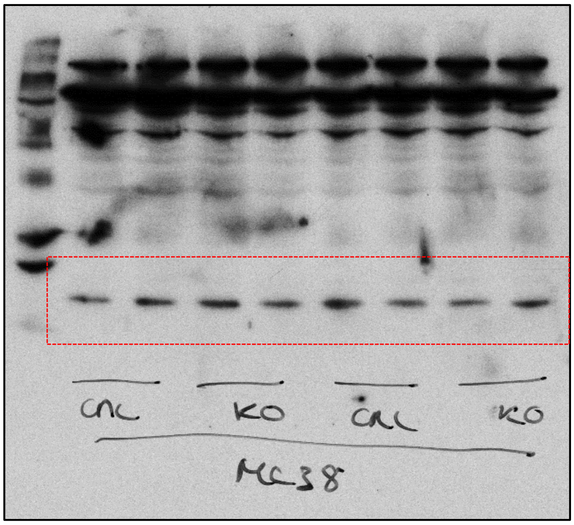

CRL IF1-KO CRL IF1-KO

β-Actin

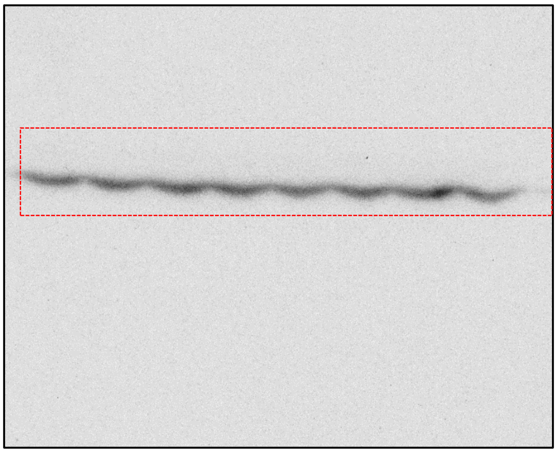

Supplemental Material to Supplemental Fig S7b (original blots)

b

E-Cadherin

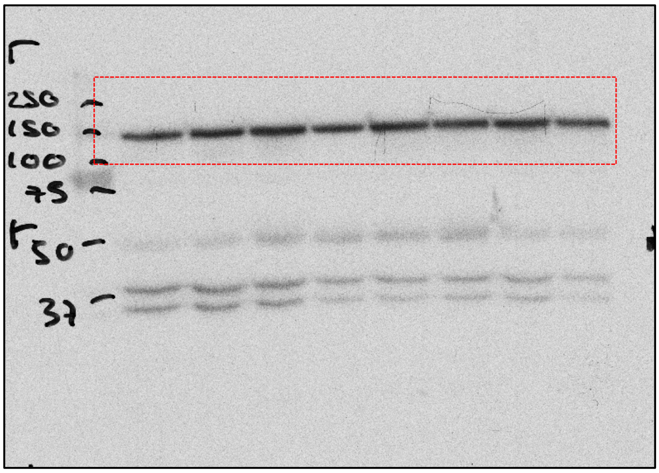

β-Actin

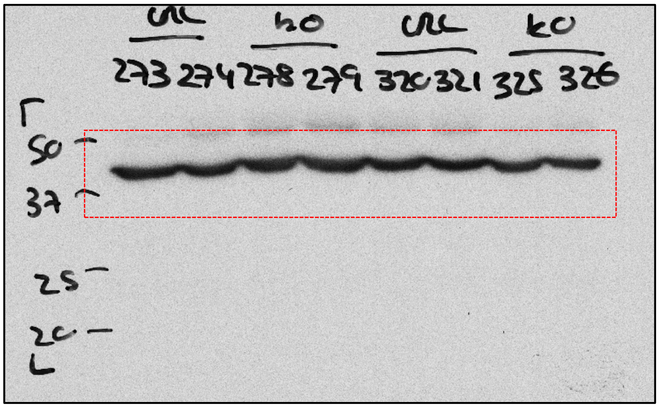

Supplement: Supplementary file 1 — Original Data File [file 41419_2023_5957_MOESM1_ESM.pdf]
