# Supplementary material for: IF1 ablation prevents ATP synthase oligomerization, enhances mitochondrial ATP turnover and promotes an adenosine-mediated pro-inflammatory phenotype
Source: Cell Death Dis. 2023 Jul 12;14(7):413. doi: 10.1038/s41419-023-05957-z (PMC10336053; doi:10.1038/s41419-023-05957-z)
Supplement: Supplementary file 2 — Supplementary Information [file 41419_2023_5957_MOESM2_ESM.docx]

**SUPPLEMENTARY INFORMATION**

**Supplementary Figure S1. Conditional knockout *Atp5if1* mouse model in intestinal epithelium. a,** Representative images of the colon of CRL and IF1-KO mice. Box-and-whisker plots show the colon length (n=4). **b,** Representative blots of the expression of processed p25 PARP1 in four independent preparations of colon from CRL and IF1-KO mice. Box-and-whisker plot shows the expression levels relative to β-Actin used as loading control (n=4). **c,** Representative blots of the expression of autophagic markers in two independent preparations of colon extracts (LC3B-I and p62) and colon mitochondria (PINK1, Parkin and Ubiquitin) from CRL and IF1-KO mice. Box-and-whisker plot shows the expression levels relative to β-Actin or β-F1 used as loading controls (n=4). **d,** Box-and-whisker plots show the expression of enzymes from proteomic data involved in peroxisomal fatty acid β-oxidation in CRL and IF1-KO mice (n=3). 2,4-dienoyl-CoA reductase, DECR2; acylcoenzyme A thioesterase 8, ACOT8; peroxisomal carnitine O-octanoyltransferase, CROT; peroxisomal 3-ketoacyl-CoA thiolase A, ACAA1A; coenzyme A peroxisomal diphosphatase, NDUT7; acylcoenzyme A peroxisomal oxidase 1, ACOX1; hydroxysteroid 17-β dehydrogenase 4, HSD17B4; enoyl-CoA hydratase and 3-hydroxyacyl CoA dehydrogenase, EHHADH. **e,** Box-and-whisker plots show the expression levels of components of OXPHOS (complex I, NDUFA9; complex II, SDH-B; complex III, Core2; complex IV, COXIV; complex V or ATP synthase, βF1) relative to Hsp60 (n=4). **f,** Representative blots of the expression of mitochondrial proteases YMEL1 and HtrA2/Omi in two independent mitochondrial preparations from CRL and IF1-KO mice. Box-and-whisker plots show the expression levels relative to Hsp60 used as loading control (n=4). *p ≤ 0.05, **p ≤ 0.01, ***p ≤ 0.001 when compared by Student's t-test. Related to data on Figure 1 and 2.

**Supplementary Figure S2. No oxidative stress in the colon of IF1 ablated mice.** **a,** Box-and-whisker plots show 8-OH-dG DNA content in total and mtDNA in CRL and IF1-KO mice (n=4). **b,** Representative blots of the expression of enzymes of cytoplasmic and mitochondrial antioxidant system in two independent preparations of colon from CRL and IF1-KO mice. Box-and-whisker plots show the corresponding quantification relative to β-Actin used as loading control (n=4). SOD1, superoxide dismutase 1; PRX2 and PRX6, peroxyredoxin 2 and 6; SOD2, superoxide dismutase 2; PRX3, peroxyredoxin 3. **c,** Representative blot of the expression of the enzymes of the mitochondrial antioxidant system in two independent preparations of colon mitochondria of CRL and IF1-KO mice. Box-and-whisker plots show the corresponding quantification relative to β-F1 used as loading control. (n=4). TRX, tiorredoxin. **d-f,** Representative blots of protein carbonylation (**d**), tyrosine nitration (**e**) and malondialdehyde modification (MDA) (**f**) of cellular proteins in four (**d**) or two (**e-f**) independent preparations of colon from CRL and IF1-KO mice. Arrowheads to the right of the blots identify the migration of the proteins used in the quantification. Histograms show the quantifications relative to β-Actin or α-Tubulin used as loading controls (n=4). Histograms represents mean ± SEM. Related to data on Figures 2.

**Supplementary Figure S3. Ablation of IF1 alters mitochondrial structure. a-d,** Box-and-whisker plots show mitochondrial cristae length (n=20-21 mitochondria) (**a**), mitochondrial electron density (n=248-250 mitochondria) (**b**), mitochondrial circularity (n=248-268 mitochondria) (**c**), number of mitochondria relative to cellular area (n=101-110 mitochondria) (**d**) in CRL and IF1-KO mice (n=4). **e,** mtDNA copy number expressed in CRL (n=5) and IF1-KO (n=4) mice. **f,** Representative blot of TFAM expression in two independent preparations of colon from CRL and IF1-KO mice. Box-and-whisker plot show the expression levels of TFAM relative to α-tubulin used as loading control (n=8). **g,** Representative blots of dynamin-related protein 1 (DRP1), optic atrophy protein 1 (OPA1), mitofusin 1 (MFN1), and 2 (MFN2) expression in two independent preparations of colon mitochondria from CRL and IF1-KO mice. Box-and-whisker plots show protein expression relative to Hsp60 used as loading control (n=8). **h,** Representative blots of mitochondrial Ca^2+^ uniporter (MCU), MCU expression regulatory subunit (MiCU), and short mitochondrial Ca^2+^-dependent ATP/Pi transporter (SCAM) expression in two independent preparations of colon mitochondria from CRL and IF1-KO mice. Box-and-whisker plots show protein expression relative to β-F1-ATPase used as loading control (n=4). **i,** Ca^2+^ retention capacity (CRC) in liver mitochondria of CRL and IF1-KO mice (n=5). Left, representative traces in the presence or absence of cyclosporine A (CsA). Right, box-and-whisker plots show the amount of Ca^2+^ required to induce PTP opening. *p ≤ 0.05, ***p ≤ 0.001 when compared by Student's t-test. Related to data on Figure 3.


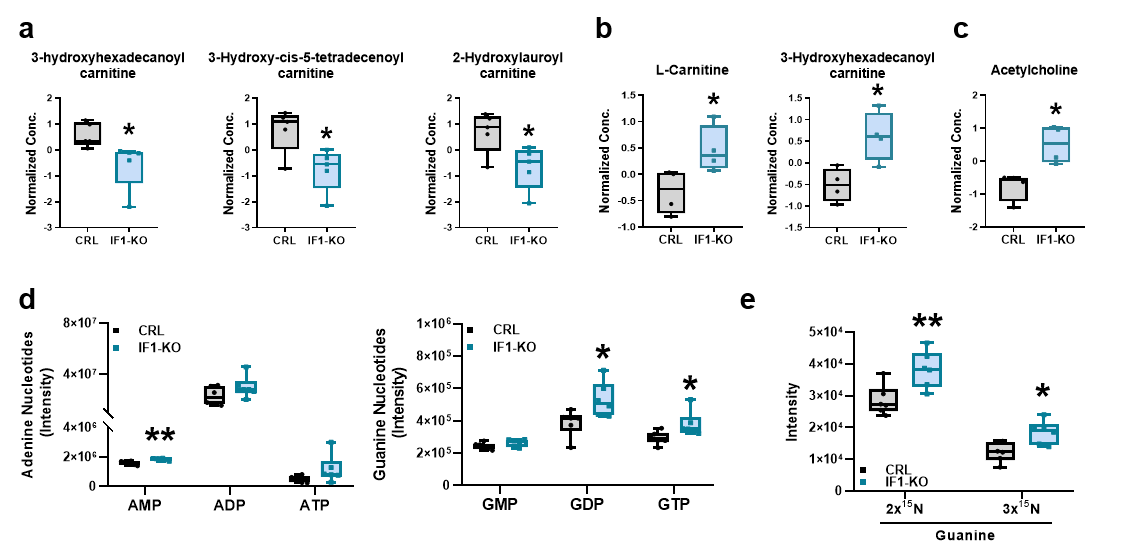


**Supplementary Figure S4. *In vivo* Ablation of IF1 increases colon fatty acid β-oxidation, acetylcholine and purine biosynthesis. a-b,** Box-and-whisker plots show metabolite levels in serum (n=5) (**a**) and in colon (n=4) (**b**) of CRL and IF1-KO mice related to fatty acid β-oxidation pathway from data of the untargeted metabolomic studies. **c,** Box-and-whisker plots show the colon levels of acetylcholine in CRL and IF1-KO mice from data of the untargeted metabolomic studies (n=4). **d,** Box-and-whisker plots show content of adenine and guanine nucleotides in colon of CRL and IF1-KO mice from data of the targeted metabolomic approach (n=6). **e,** Box-and-whisker plots show the levels of nucleotides incorporating two or three ^15^N after a pulse with ^15^N-glutamine in colon of CRL and IF1-KO mice (n=6). *p ≤ 0.05, **p ≤ 0.01 when compared by Student's t-test. Related to data on Figure 4.

**
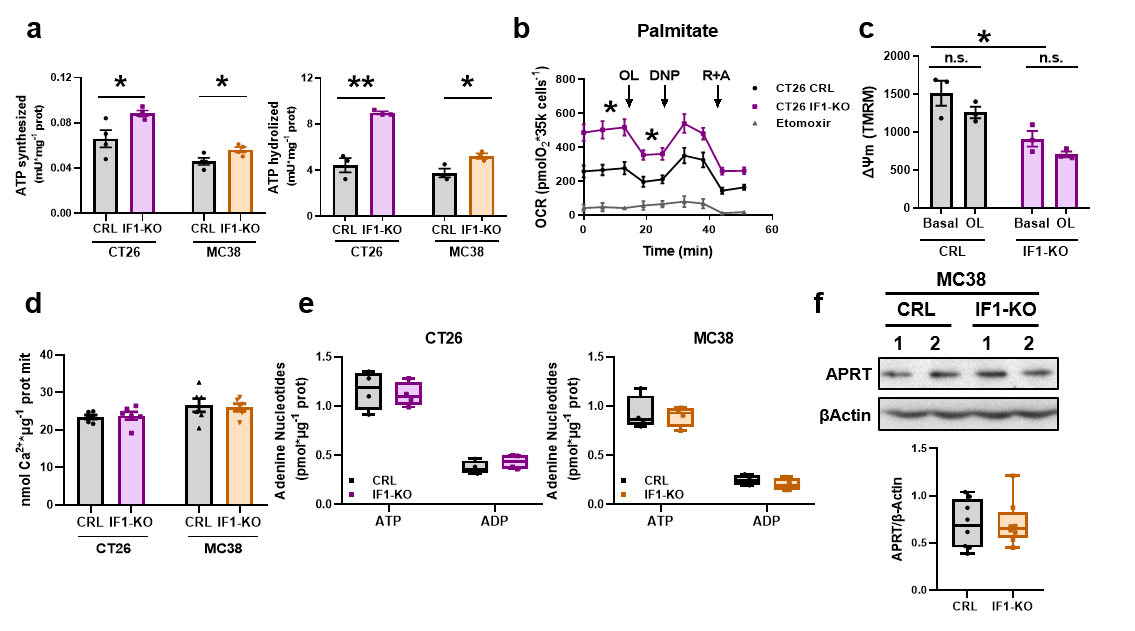
**

**Supplementary Figure S5. IF1 loss-of-function cellular models recapitulate IF1-KO mice phenotype. a,** ATP synthetic and hydrolytic activity of ATP synthase in CT26 and MC38 CRL and IF1-KO cells (n=3-4). **b,** Seahorse XF24 oxygen consumption rate (OCR) profiles of CT26 CRL and IF1-KO cells (n=4) in the presence of palmitate as respiratory substrate. **c,** Histograms show mitochondrial membrane potential (∆Ψm) in CT26 CRL and IF1-KO cells (n=3). The effect of oligomycin (OL)-is also shown. **d,** Histograms show the intramitochondrial Ca^2+^ content of isolated mitochondria from CT26 and MC38 CRL and IF1-KO cells (n=6). **e,** Box-and-whisker plots show ATP and ADP content in CT26 and MC38 CRL and IF1-KO cells (n=4). **f,** Representative blots show the expression of adenine phosphoribosyl transferase (APRT) in two independent sample preparation of MC38 CRL and IF1-KO cells. Box-and-whisker plots show the corresponding quantitation relative to β-Actin used as loading control (n=4). *p ≤ 0.05, **p ≤ 0.01 when compared by Student's t-test. Related to data on Figure 5.

**
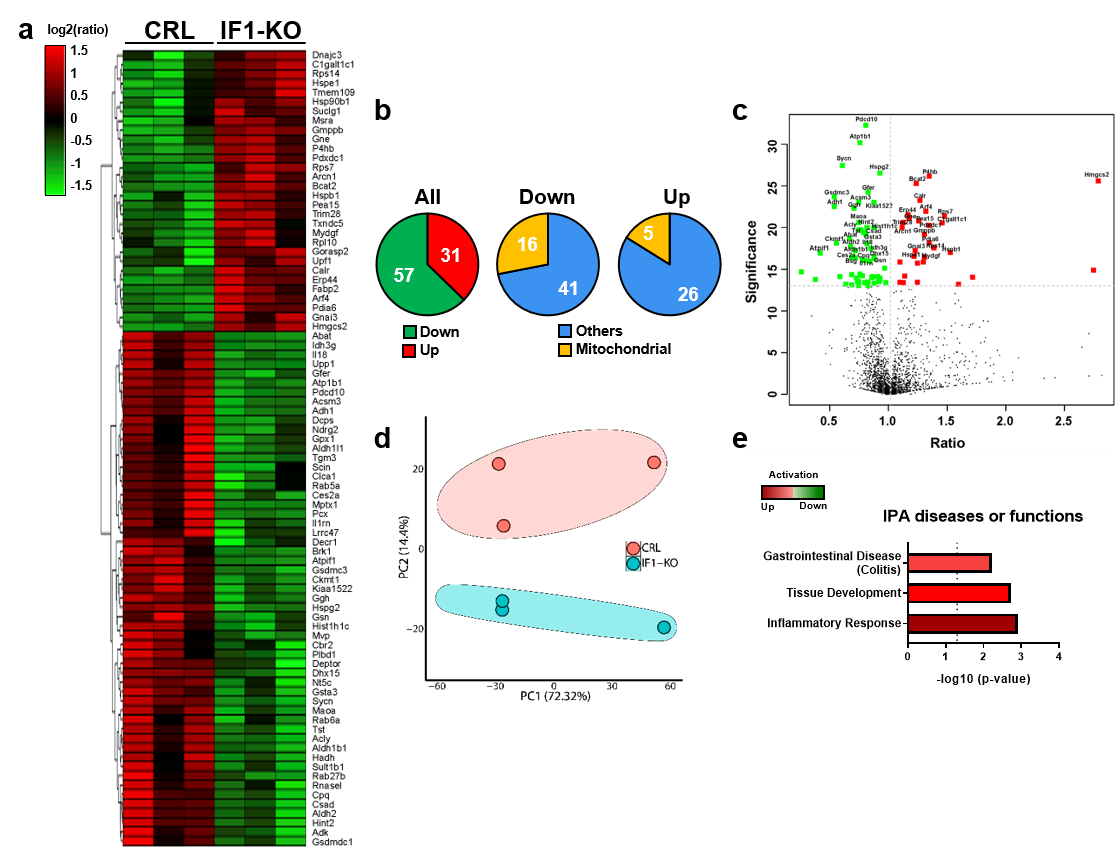
**

**Supplementary Figure S6. Proteome and signaling pathways in the colon of IF1-KO mice.** iTRAQ proteomic analysis of colon from control (CRL) (n=3) and IF1-KO (n=3) mice. **a,** Heat Map representation showing decreased (green) and increased (red) proteins in colon of IF1-KO mice. **b,** Diagram representing the total amount of proteins increased or decreased in colon of IF1-KO mice. Middle and right diagrams showing differentially expressed proteins as a function of their cellular location. **c,** Volcano plot showing differentially expressed proteins between the two genotypes. **d,** Principal component (PC) analysis showing the two genotypes. **e,** Ingenuity Pathway Analysis (IPA) of the predicted activation or inhibition of different diseases or functions in colon of IF1-KO mice. Related to data on Figure 6.


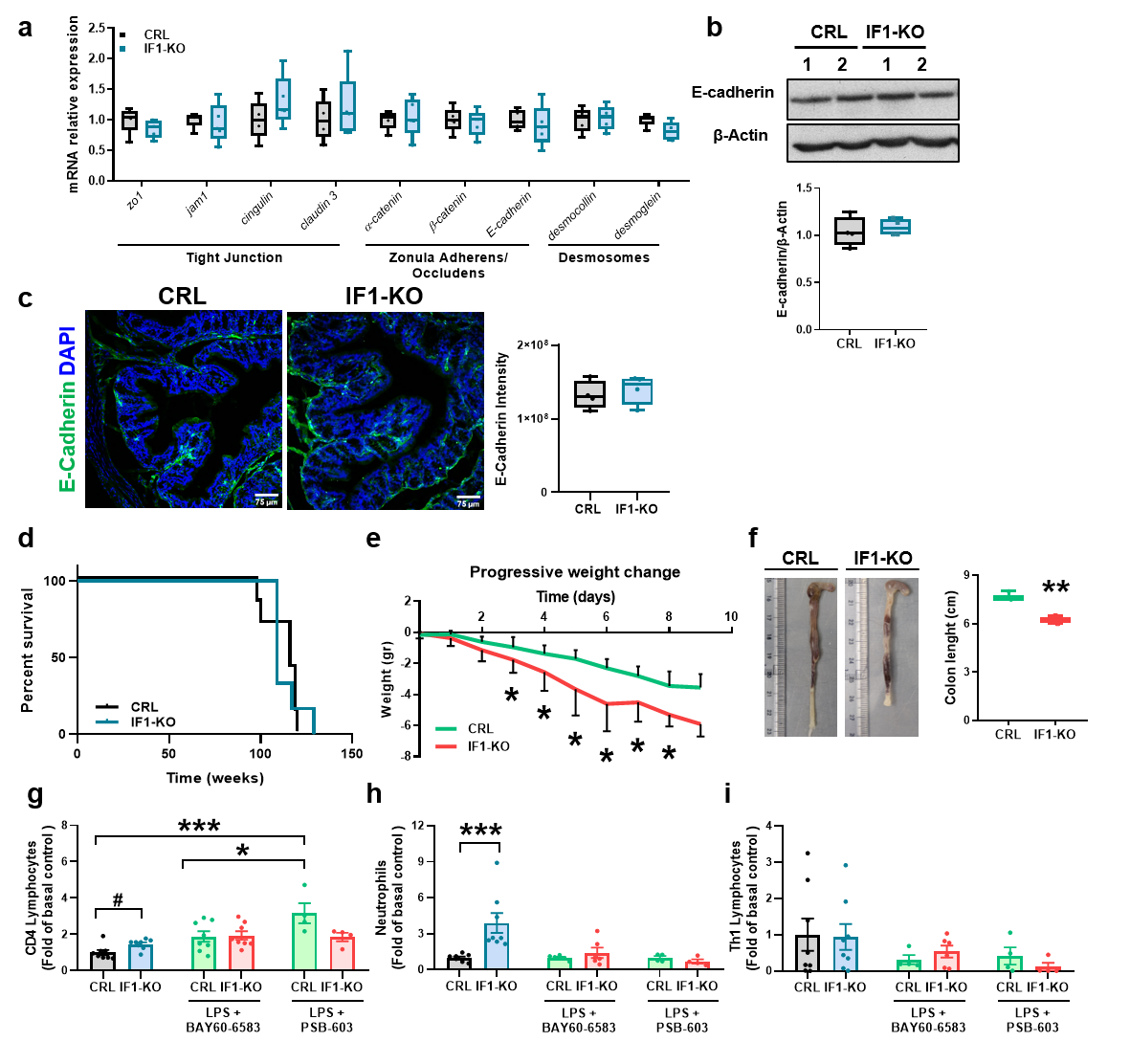


**Supplementary Figure S7. The permeability of the intestinal barrier is altered due to IF1 ablation. a,** mRNA expression levels of the relevant proteins involved the intestinal epithelial barrier in CRL and IF1-KO mice (n=5). **b,** Representative blot of E-cadherin expression in two independent preparations of CRL and IF1-KO mice. Box-and-whisker plots show the corresponding quantification relative to β-Actin used as loading control (n=4). **c,** Representative immunofluorescence images showing the expression of E-cadherin (green) in CRL (n=3-4) and IF1-KO (n=4) mice. Box-and-whisker plots show the corresponding quantification. DAPI (blue) stained nuclei. **d,** Kaplan-Meier survival analysis of CRL (n=6) and IF1-KO (n=7) mice. **e,** Progressive weight change (mean ±SEM) of CRL (n=8) and IF1-KO (n=9) mice treated with 2% DSS for ten days. **f,** Representative images of the colon of CRL and IF1-KO mice (n=3) after six days of 2% DSS treatment. Box-and-whisker plots show the colon length. **g-i,** Histogram show the percentage of CD4^+^ lymphocytes (CD45^+^ CD4^+^ DAPI^-^) (**g**), neutrophils (CD45^+^ CD11b^+^ Ly-6G^+^ DAPI^-^) (**h**) and Th1 (CD4^+^ IFNγ^+^) (**i**) in the colon of CRL and IF1-KO mice non-treated (n=8) or treated with LPS and the agonist BAY60-6583 (n=6-8) or antagonist PSB-603 (n=4). Histograms represent the mean ±SEM. For panels **a-f,** *p ≤ 0.05, **p ≤ 0.01 when compared by Student's t-test. For panels **g-i,** *p ≤ 0.05, **p ≤ 0.01, ***p ≤ 0.01 when compared by one-way ANOVA test and the Tukey multiple correction test. #p ≤ 0.05 when compared by Student's t-test. Related to data on Figures 6 and 7.

**Supplementary Table S1. Oligonucleotides used in this study.**

| Name | Sequence | Source |
| --- | --- | --- |
| ATPIF1-F | 5’-TGCCTGACATTGGTATTGGG-3’ | Mouse Genetics Project  (Wellcome Trust Sanger Institute) |
| ATPIF1-R | 5’-GTGCAGCTTGTGGGAGTCAG-3’ | Mouse Genetics Project  (Wellcome Trust Sanger Institute) |
| VillCre-F | 5’- CAAGCCTGGCTCGACGGCC -3’ | Mouse Genetics Project  (Wellcome Trust Sanger Institute) |
| VillCre-R | 5’- CGCGAACATCTTCAGGTTCT -3’ | Mouse Genetics Project  (Wellcome Trust Sanger Institute) |
| Atp5f1a-F | 5’-TTCAAGCAGAGGAGATGGTG-3’ | IDT |
| Atp5f1a-R | 5’-CATTGTCGGGTTCCAAGTTC-3’ | IDT |
| Atp5f1b-F | 5’-TCCACTGGACTCCACCTCTC-3’ | IDT |
| Atp5f1b-R | 5’-CCGTGAAGACCTCAGCAACT-3’ | IDT |
| Atp5f1c-F | 5’-CCGAGTGTATGGGACAGGTT-3’ | IDT |
| Atp5f1c-R | 5’-GCACCACAAAGCCCTCTATC-3’ | IDT |
| Atp5if1-F | 5’-CGGACTCGTCGGATAGCA-3’ | IDT |
| Atp5if1-R | 5’-CTCCTTCGAATGGTGGTCAA-3’ | IDT |
| Atp5mc1-F | 5’-GGCACAGTGTTTGGTAGCTTG-3’ | IDT |
| Atp5mc1-R | 5’-CCCAGAATGGCATAGGAGAA-3’ | IDT |
| Atp5pd-F | 5’-ATGCCCTGAAGATTCCTGTG-3’ | IDT |
| Atp5pd-R | 5’-TCCAGCTGCTTCTCATACTCC-3’ | IDT |
| Cingulina-F | 5’-GCAGGACAGGCTGAGCTTAC-3’ | IDT |
| Cingulina-R | 5’-CTCTTCAGCCTTCTCCTCCA-3’ | IDT |
| Claudina-3-F | 5’-CTCTTCAGCCTTCTCCTCCA-3’ | IDT |
| Claudina-3-R | 5’-GGCTGAGCTTACCCGAAAA-3’ | IDT |
| Cox4i1-F | 5’-GAGAGCCATTTCTACTTCGGT-3’ | IDT |
| Cox4i1-R | 5’-GCAGACAGCATCGTGACAT-3’ | IDT |
| Cox5a-F | 5’-CATCCAGGAACTTAGACCAACT-3’ | IDT |
| Cox5a-R | 5’-TTTCCAGGCAACTGTTTCAATC-3’ | IDT |
| Cox8a-F | 5’-CATCTTGACTCCCTGACCTTG-3’ | IDT |
| Cox8a-R | 5’-CTTCGAGTGGACCTGAGC-3’ | IDT |
| Desmocollina-F | 5’-CACTACCACGTCGGCTCA-3’ | IDT |
| Desmocollina-R | 5’-TGGGCAGGTTGTCATTCA-3’ | IDT |
| Desmogleina-F | 5’-TCTTGACCGGGAAGAAACAC-3’ | IDT |
| Desmogleina-R | 5’-GTGTGAACACTGGCTCGTTG-3’ | IDT |
| Dnm1l-F | 5’-GCAACATCAGAAGCACTCAAG-3’ | IDT |
| Dnm1l-R | 5’-AACCCTTCCCATCAATACATCC-3’ | IDT |
| E-cadherina-F | 5’-GACAGAAACGAGACTGGGTCA-3’ | IDT |
| E-cadherina-R | 5’-GCCGGTGATGCTGTAGAAAA-3’ | IDT |
| Etfdh-F | 5’-CGCCTCTCTCCTTTGTATCTG-3’ | IDT |
| Etfdh-R | 5’-CTTGGAGCACACAGAGGTAG-3’ | IDT |
| GADPH-F | 5’-TGCGACTTCAACAGCAACTC-3’ | IDT |
| GAPDH-R | 5’-GGATAGGGCCTCTCTTGCTC-3’ | IDT |
| Immt-F | 5’-GTGGCACAGAGCCAGAAAA-3’ | IDT |
| Immt-R | 5’-CCGGGACCGAAAGTGTATC-3’ | IDT |
| JAM1-F | 5’-ATGGTCTCCGAGGAAGGTG-3’ | IDT |
| JAM1-R | 5’-GACAGAGGAGGGGACACTGA-3’ | IDT |
| Mic10-F | 5’-GAAGCTAGGTACTGGGTTTGGA-3’ | IDT |
| Mic10-R | 5’-GCCAGAACCAAAGGCTAATG-3’ | IDT |
| mt12S-F | 5’-AAACAGCTTTTAACCATTGTAGGC-3’ | IDT |
| mt12S-R | 5’-TTGAGCTTGAACGCTTTCTTTA-3’ | IDT |
| mt16S-F | 5’-CACTGCCTGCCCAGTGA-3’ | IDT |
| mt16S-R | 5’-ATACCGCGGCCGTTAAA-3’ | IDT |
| mtND4-F | 5’-AACGGATCCACAGCCGTA-3’ | IDT |
| mtND4-R | 5’-AGTCCTCGGGCCATGATT-3’ | IDT |
| nActb-F | 5’-ACCCAGAGAGCTCACCATTC-3’ | IDT |
| nActb-R | 5’-TGATCCACATCTGCTGGAAG-3’ | IDT |
| nAtp5b-F | 5’-CCTGCTGATGACCTGACTGA-3’ | IDT |
| nAtp5b-R | 5’-GCTGGATAGATGCCCAACTC-3’ | IDT |
| nB2M-F | 5’-CCGGATTGGCTGTGAGTT-3’ | IDT |
| nB2M-R | 5’-GACAAGCACCAGAAAGACCAG-3’ | IDT |
| Nd1-F | 5’-GCCAGGAAATTGCGTAAGAC-3’ | IDT |
| Nd1-R | 5’-TAGAATGGGGACGAGGAGTG-3’ | IDT |
| Nd5-F | 5’-TGATGGTACGGACGAACAGA-3’ | IDT |
| Nd5-R | 5’-CTCCGATGCGGTTATAGAGG-3’ | IDT |
| Ndufa10-F | 5’-TCCTGGAGGCAATGTACAAC-3’ | IDT |
| Ndufa10-R | 5’-TCGATATAGATGACTGCGTGTG-3’ | IDT |
| Ndufa9-F | 5’-GCACGAGACAAAGATTCTATCAG-3’ | IDT |
| Ndufa9-R | 5’-TCACAAAAACATCCTCAAAATCAAAG-3’ | IDT |
| Ndufs3-F | 5’-ATCCTGACAGATTATGGCTTCG-3’ | IDT |
| Ndufs3-R | 5’-CACTACCCGCTTTACCTCATC-3’ | IDT |
| Ndufs7-F | 5’-GGCTACTACCACTACTCCTACT-3’ | IDT |
| Ndufs7-R | 5’-CTTCTGTTCACGCTTGATCTTC-3’ | IDT |
| Oma1-F | 5’-GCAAAGAACACTTCCGACTTC-3’ | IDT |
| Oma1-R | 5’-TTCTTTCACAGTCAGGTAGCG-3’ | IDT |
| Opa1-F | 5’-AGCATTTCGAGCAACAGATCA-3’ | IDT |
| Opa1-R | 5’-CGCTCCAAGATCCTCTGATAC-3’ | IDT |
| Sdha-F | 5’-TCCATACACCGAATAAGAGCAAA-3’ | IDT |
| Sdha-R | 5’-ACCAGCCCTAGTGACCAT-3’ | IDT |
| Sdhb-F | 5’-CTGTACGAGTGCATCCTGTG-3’ | IDT |
| Sdhb-R | 5’-TCCTCTGTGAAGTCGTCTCT-3’ | IDT |
| Timm22-F | 5’-AAGAACAGCGTCATCAGTGG-3’ | IDT |
| Timm22-R | 5’-CAGCAGAGAAAGCAGCAA-3’ | IDT |
| Timm23-F | 5’-GATGACCTCAACACAGTAGCAG-3’ | IDT |
| Timm23-R | 5’-GTGCATAGAGACTGGTGAGTG-3’ | IDT |
| Tomm20-F | 5’-TCCTTGAAGAGATACAGCTTGG-3’ | IDT |
| Tomm20-R | 5’-CAGCAATGGCATTTGTCAGG-3’ | IDT |
| Tomm40-F | 5’-GAGCAACCGTTTCCAGGT-3’ | IDT |
| Tomm40-R | 5’-CACTATTGTCCATGTCACCCA-3’ | IDT |
| Uqcrc1-F | 5’-GCCACTATGACTGCACTTACG-3’ | IDT |
| Uqcrc1-R | 5’-GCCCAGTATCAGAGTAGGAGA-3’ | IDT |
| Uqcrc2-F | 5’-GCTAATCCTTTGTACTGTCCTGA-3’ | IDT |
| Uqcrc2-R | 5’-AGACAGAATGACTCACACCAAG-3’ | IDT |
| Yme1l1-F | 5’-GTATTCAAGGCAGACGATCAATC-3’ | IDT |
| Yme1l1-F | 5’-GACCAGGACGTATTAAGGCATT-3’ | IDT |
| ZO-1-F | 5’-CAAAGAGATGAGCGGGCTAC-3’ | IDT |
| ZO-1-R | 5’-AGCGACCTGAATGGTCTGAT-3’ | IDT |
| α-catenina-F | 5’-GCTGCTGATTCTGGCTGAC-3’ | IDT |
| α-catenina-R | 5’-GTTCATTGCCAGCATTCCTC-3’ | IDT |
| β-actina-F | 5’-AACACAGTGCTGTCTGGTGGT-3’ | IDT |
| β-actina-R | 5’-GATCCACATCTGCTGGAAGG-3’ | IDT |
| β-catenina-F | 5’-CTATCAGGATGACGCGGAAC-3’ | IDT |
| β-catenina-R | 5’-CATGATGGCATGTCTGGAAG-3’ | IDT |

**Supplementary Table S2. Antibodies used in this study.**

| **Antibodies** | **Reference or Source** | **Identifier or Catalog Number** |
| --- | --- | --- |
| Rabbit anti-IF1 (1:1,000) | José M. Cuezva (Esparza-Moltó et al., 2019) | N/A |
| Mouse anti-β-F1 (clone 11/21-7A8) (1:1,000) | José M. Cuezva (Acebo et al., 2009) | N/A |
| Mouse anti-γ-F1 | José M. Cuezva [[1](#_ENREF_1)] | N/A |
| Rabbit anti-Ki67 (1:250) | Thermo Fisher | Cat# MA1-90584 |
| Rabbit anti-c-casp3 (clone D175) (1:500) | Cell Signaling | Cat# 9661; RRID: AB_2341188 |
| Rabbit anti-Cleaved PARP1 p25 (1:1,000) | ABclonal | Cat# A19612 |
| Rabbit anti-β-F1 (1:20,000) | José M. Cuezva  [[2](#_ENREF_2)] | N/A |
| Mouse anti-G6PDH (1:1,000) | Thermo Fisher | Cat# PA1-84814; RRID:AB_2107525 |
| Mouse anti-GAPDH (clone 273A-E5) (1:1,000) | José M. Cuezva [[3](#_ENREF_3)] | N/A |
| Rabbit anti-PK (1:1,000) | Abcam | Cat# ab38237; RRID:AB_777576 |
| Mouse anti-LDHA (clone 4D3-A1) (1:1,000) | José M. Cuezva [[4](#_ENREF_4)] | N/A |
| Rabbit anti-LC3B (1:1,000) | Cell Signaling | Cat# 4108; RRID: AB_2137703 |
| Rabbit anti-p62 (1:1,000) | Enzo Life Sciences | Cat# BML-PW9860; RRID: AB_2196009 |
| Rabbit anti-PINK1 (1:1,000) | Abcam | Cat# ab23707; RRID: AB_447627 |
| Rabbit anti-Parkin (1:1,000) | Abcam | Cat# ab77924; RRID: AB_1566559 |
| Rabbit anti-Ubiquitin (1:1,000) | MilliporeSigma | Cat# 662099-50UL; RRID: AB_565250 |
| Mouse anti-NADHs9 (NDUFA9, clone 15/22-5) (1:1,000) | José M. Cuezva [[4](#_ENREF_4)] | N/A |
| Mouse anti-UQCRC2 (clone 13G12AF12BB11) (1:1,000) | Abcam | Cat# ab14745; RRID: AB_2213640 |
| Mouse anti-SDH-B (clone 21A11AE7) (1:1,000) | Invitrogen | Cat# 459230; RRID: AB_2532233 |
| Mouse anti-COX IV (clone 20E8C12) (1:1,000) | Abcam | Cat# ab14744; RRID: AB_301443 |
| Mouse anti-HSP60 (clone 17/9-15 G1) (1:5,000) | José M. Cuezva [[3](#_ENREF_3)] | N/A |
| Rabbit anti-HtrA2/Omi (1:250) | Cell Signaling | Cat# 9745; RRID: AB_11220423 |
| Rabbit anti-YME1L1 (1:250) | Thermo Fisher (PA5-24808) | Cat# PA5-24808;RRID: AB_2542308 |
| Rabbit anti-SOD1 (1:1,000) | Santa Cruz Biotech. | Cat# sc-11407; RRID: AB_2193779 |
| Rabbit anti-PRX2 (1:1,000) | Abcam | Cat# ab109367; RRID: AB_10862524 |
| Rabbit anti-PRX6 (1:1,000) | Abcam | Cat# ab59543; RRID: AB_944762 |
| Rabbit anti-SOD2 (1:1,000) | Santa Cruz Biotech. | Cat# sc-30080; RRID: AB_661470 |
| Rabbit anti-PRX3 (1:1,000) | Abcam | Cat# ab222807 |
| Rabbit anti-TRX (1:1,000) | Cusabio | Cat # PA02814A0Rb |
| Mouse anti-β-actin (clone AC-74) (1:10,000) | MilliporeSigma | Cat# A5316; RRID: AB_476743 |
| Mouse anti-3-Nitrotyrosine (clone 39B6) (1:1,000) | Abcam | Cat# ab61392; RRID: AB_942087 |
| Mouse anti-4-Hydroxynonenal (clone 198960) (1:1,000) | R&D Systems | Cat# MAB3249; RRID: AB_664165 |
| Rabbit anti-Malondialdehyde (1:1,000) | Abcam | Cat# ab27642; RRID: AB_776164 |
| Rabbit anti-TFAM (1:500) | Abcam | Cat# ab47517; RRID: AB_945799 |
| Mouse anti-α-tubulin (clone DM1A) (1:10,000) | MilliporeSigma | Cat# T9026; RRID: AB_477593 |
| Mouse anti-DLP1 (DRP1, clone 18/DLP1) (1:1,000) | BD Biosciences | Cat# 611112; RRID: AB_398423 |
| Mouse anti-OPA1 (clone 18/OPA1) (1:1,000) | BD Biosciences | Cat# 612606; RRID: AB_399888 |
| Rabbit Anti-MFN1 (clone EPR7960) (1:1,000) | Abcam | Cat# ab129154; RRID: AB_11142211 |
| Mouse anti-MFN2 (clone XX-1) (1:1,000) | Santa Cruz Biotech. | Cat# sc-100560; RRID: AB_2235195 |
| Mouse anti-MIC60 (clone 2E4AD5) (1:1,000) | Abcam | Cat# ab110329; RRID: AB_2533824 |
| Rabbit anti-MCU (1:1,000) | MilliporeSigma | Cat# HPA016480; RRID:A B_2071893 |
| Rabbit anti-MiCU (1:1,000) | MilliporeSigma | Cat# HPA037480; RRID: AB_10696934 |
| Rabbit anti-SCaMC-1 (1:5,000) | Jorgina Satrústegui  [[5](#_ENREF_5)] | N/A |
| Rabbit anti-APRT (1:1,000) | ABclonal | Cat# A5456; RRID. AB_2766257 |
| Mouse anti-E-cadherin (clone 36/E) (1:500) | BD Biosciences | Cat# 610181; RRID: AB_397580 |
| Rabbit anti-CD4 (clone EPR19514) (1:500) | Abcam | Cat# ab183685; RRID: AB_2686917 |
| Mouse anti-FOXP3 (clone 236A/E7) (1:300) | Abcam | Cat# ab20034; RRID: AB_445284 |
| Rat anti-F4/80 (clone Cl:A3-1) (1:200) | Bio-Rad | Cat# MCA497RT; RRID: AB_1102558 |
| Rat anti-CD8 (clone 53-6.7) (1:100) | Invitrogen | Cat# MA1-10303; RRID: AB_11153636 |
| Mouse anti-RORγt (clone Q31-378) (1:100) | BD Biosciences | Cat# 563081; RRID: AB_2686896 |
| Mouse anti-CD3 (clone 145-2C11) (1:100) | Invitrogen | Cat# 25-0031-82; RRID: AB_1148909 |
| Rabbit anti-CD4 (clone RM4-5) (1:100) | Invitrogen | Cat# 11-0042-82; RRID: AB_464896 |
| Mouse anti-CD45 (clone 30-F11) (1:100) | Invitrogen | Cat# 17-0451-82; RRID: AB_469392 |
| Rat anti-CD8 (clone 53-6.7) (1:100) | BioLegend | Cat# 100713; RRID: AB_470429 |
| Mouse anti-CD11d (clone M1/70) (1:100) | Invitrogen | Cat# 45-0112-82; RRID: AB_953558 |
| Mouse anti-IFNγ (clone XMG1.2) (1:100) | BioLegend | Cat# 505830; RRID: AB_2563105 |
| Mouse anti-IL-17A (clone eBio17B7) (1:100) | Invitrogen | Cat# 17-7177-81; RRID: AB_2659804 |
| Mouse anti-Ly-6G (clone 1A8) (1:100) | BioLegend | Cat# 127607; RRID: AB_1186104 |
| Mouse anti-Ly-6C (clone HK1.4) (1:100) | BioLegend | Cat# 128005; RRID: AB_2830201 |
| Peroxidase-conjugated rabbit anti-mouse IgGs (1:5,000) | Nordic Immunology | Cat# RAM/IgG(H+L)/PO |
| Peroxidase-conjugated goat anti-rabbit IgGs (1:5,000) | Nordic Immunology | Cat# GAR/IgG(H+L)/PO |
| Alexa 488-conjugated donkey anti-rabbit IgGs (1:500) | Thermo Fisher | Cat# A-21206; RRID: AB_2535792 |
| Alexa 488-conjugated donkey anti-rat IgGs (1:500) | Thermo Fisher | Cat# A-21208; RRID: AB_2535794 |
| Alexa 555-conjugated donkey anti-rabbit IgGs (1:500) | Thermo Fisher | Cat# A-31572; RRID: AB_162543 |
| Alexa 555-conjugated donkey anti-mouse IgGs (1:500) | Thermo Fisher | Cat# A-31570; RRID: AB_2536180 |
| Alexa 555-conjugated goat anti-rat IgGs (1:500) | Thermo Fisher | Cat# A-21434; RRID: AB_2535855 |

**References**

1. Willers IM, Martínez-Reyes I, Martínez-Diez M, Cuezva J. miR-127-5p targets the 3'UTR of human β-F1-ATPase mRNA and inhibits its translation. Biochim Biophys Acta-Bioenergetics. 2012;1817(5):838-48.

2. Cuezva JM, Krajewska M, de Heredia ML, Krajewski S, Santamaria G, Kim H, et al. The bioenergetic signature of cancer: a marker of tumor progression. Cancer Res. 2002;62(22):6674-81.

3. Acebo P, Giner D, Calvo P, Blanco-Rivero A, Ortega AD, Fernandez PL, et al. Cancer abolishes the tissue type-specific differences in the phenotype of energetic metabolism. Transl Oncol. 2009;2(3):138-45.

4. Santacatterina F, Chamorro M, Nuñez de Arenas C, Navarro C, Martin MA, Cuezva JM, et al. Quantitative analysis of proteins of metabolism by reverse phase protein microarrays identifies potential biomarkers of rare neuromuscular diseases. J Trans Med. 2015;13:65.

5. del Arco A, Satrustegui J. Identification of a novel human subfamily of mitochondrial carriers with calcium-binding domains. J Biol Chem. 2004;279(23):24701-13.
