# Supplementary material for: IF1 ablation prevents ATP synthase oligomerization, enhances mitochondrial ATP turnover and promotes an adenosine-mediated pro-inflammatory phenotype
Source: Cell Death Dis. 2023 Jul 12;14(7):413. doi: 10.1038/s41419-023-05957-z (PMC10336053; doi:10.1038/s41419-023-05957-z)
Supplement: Supplementary file 4 — filled CDD author contribution form [file 41419_2023_5957_MOESM4_ESM.pdf]

DECLARATION OF CONTRIBUTIONS TO ARTICLE **ADMC**

Manuscript Number:	Journal Name:	
	Cell Death & Differentiation	(the 'Journal')

Proposed Title of the Contribution:

IF1 ablation prevents ATP synthase oligomerization, enhances mitochondrial ATP turnover and promotes an adenosine-mediated pro-inflammatory phenotype	(the 'Contribution)

Author(s):

Sonia Domínguez-Zorita, Inés Romero-Carramiñana, Fulvio Santacatterina, Pau B. Esparza-Moltó, Carolina Simó, Araceli del-Arco, Cristina Núñez de Arenas, Jorge Saiz, Coral Barbas and José M. Cuezva	(the 'Authors')

For all *CDD* articles, each person named as an author in the published version must be able to show he or she has contributed substantially to the article.

Authorship credit should be based on 1) substantial contributions to conception and design, acquisition of data, or analysis and interpretation of data; 2) drafting the article or revising it critically for important intellectual content; and 3) final approval of the version to be published. Authors should meet conditions 1, 2 and 3.

Any person who cannot be shown to have made a substantial contribution to the article cannot be listed as an author in the final version. The name of any person who is deemed to have made a minor contribution can, however, appear in the Acknowledgments section of the article.

Please complete the table below to indicate the contributions of all named authors to the manuscript.

Author Full Name:	Specification of Contribution to the Manuscript:
Sonia Domínguez-Zorita	SDZ carried out experiments, processed the experimental data, performed the analysis, drafted the manuscript and designed the figures.
Inés Romero-Carramiñana	IRC carried out experiments, processed the experimental data, performed the analysis,
Fulvio Santacatterina	FS carried out experiments, processed the experimental data, performed the analysis,
Pau B. Esparza-Moltó	PBEM carried out experiments, processed the experimental data, performed the analysis,
Carolina Simó	CS carried out experiments related to untargeted metabolomic analyses (Figure 4). CS analyze them and plot the results.
Araceli del-Arco	ADA carried out the experiments on cell lines related to the intramitochondrial ATP turnover.
Cristina Núñez de Arenas	CNA manage the project in terms of the mouse models.
Jorge Saiz	JS carried out experiments related to targeted metabolomic analyses and analyze them (Figure 4).
Coral Barbas	CB carried out experiments related to targeted metabolomic analyses and analyze them (Figure 4).
José M. Cuezva	JMC conceived the proyect, designed and analyzed experiments, designed figures, secured funding and wrote the manuscript.

Please complete the table below to indicate the contributions of all named authors to the figures.

Figure 1:

a-c) PBEM developed IF1-floxed mice.
a-d) SDZ and FS.
e-i) SDZ.
Supplementary Fig. S1:
a) FS.
b-d) SDZ.

Figure 2:

a-i) SDZ.
i) IRC.

Figure 3:

a-b) SDZ and FS.
c-i) SDZ.
Supplementary Fig. S2:
a-i) SDZ.

Figure 4:

a-e, g) CS.
f, i-k) SDZ.
h) JS, CB.
Supplementary Fig. S3:
a-c) CS.
d-e) JS and CB.

Figure 5:

a-b; d-k) SDZ.
c) IRC.
j-k) ADA.
Supplementary Fig. S4:
a-f) SDZ and IRC.

Figure 6:

a) SDZ.
b-d) FS.
e-k) FS and SDZ.
Supplementary Fig. S5:
a-e) SDZ and FS.
Supplementary Fig. S6:
a, g-i) SDZ and IRC.
b-d) SDZ.
e-f) SDZ and FS.
Figure 7:
IRC and SDZ.

Signed for and on behalf of the Author(s):	Print Name:	Date:
JMC	José M. Cuezva	19/01/2023
